# DeXtrusion: automatic recognition of epithelial cell extrusion through machine learning *in vivo*

**DOI:** 10.1242/dev.201747

**Published:** 2023-06-30

**Authors:** Alexis Villars, Gaëlle Letort, Léo Valon, Romain Levayer

**Affiliations:** Department of Developmental and Stem Cell Biology, Institut Pasteur, Université de Paris Cité, CNRS UMR 3738, 25 rue du Dr. Roux, 75015 Paris, France

**Keywords:** Deep learning, Convolutional neural network, Epithelia, Live imaging, Extrusion, Cell division, SOPs, *Drosophila*

## Abstract

Accurately counting and localising cellular events from movies is an important bottleneck of high-content tissue/embryo live imaging. Here, we propose a new methodology based on deep learning that allows automatic detection of cellular events and their precise *xyt* localisation on live fluorescent imaging movies without segmentation. We focused on the detection of cell extrusion, the expulsion of dying cells from the epithelial layer, and devised DeXtrusion: a pipeline based on recurrent neural networks for automatic detection of cell extrusion/cell death events in large movies of epithelia marked with cell contour. The pipeline, initially trained on movies of the *Drosophila* pupal notum marked with fluorescent E-cadherin, is easily trainable, provides fast and accurate extrusion predictions in a large range of imaging conditions, and can also detect other cellular events, such as cell division or cell differentiation. It also performs well on other epithelial tissues with reasonable re-training. Our methodology could easily be applied for other cellular events detected by live fluorescent microscopy and could help to democratise the use of deep learning for automatic event detections in developing tissues.

## Teaser

**Summary:** A new and fully available method based on machine learning can detect automatically cellular events (including cell extrusion, cell division and cell differentiation) from fluorescent movies of epithelia *in vivo*.

## INTRODUCTION

Epithelial tissues can be dramatically remodelled during embryogenesis or in adult organs undergoing fast turnover. These events are often associated with high rates of cell elimination, which requires fine control of the absolute number of dying cells, as well as their distribution in time and space. Cell extrusion is a sequence of remodelling steps leading to apical constriction and cell elimination from the epithelial layer without impairing the sealing properties of the tissue (Rosenblatt et al., 2001). This process is highly coordinated between the extruding cell and its neighbours: as the cell extrudes its neighbours are brought close to each other to maintain epithelial sealing and stability (Villars and Levayer, 2022).

The tight spatiotemporal control of epithelial cell apoptosis plays an essential role during tissue morphogenesis (Ambrosini et al., 2017). For instance, a spatial bias in the distribution of cell death can locally modulate growth and final tissue shape (Matamoro-Vidal et al., 2022 preprint); apoptosis and cell extrusions can generate local traction forces to fuse tissues (Toyama et al., 2008), promote local tissue bending (Monier et al., 2015; Roellig et al., 2022) or can be permissive for global tissue remodelling through the modulation of tissue viscosity (Ranft et al., 2010; Suzanne et al., 2010). Moreover, tight regulation of the precise spatiotemporal distribution of cell extrusion/cell death is also essential to maintain the cohesion of the tissue, especially in conditions with high rates of cell elimination (Valon et al., 2021). These examples all rely on the precise regulation of the number and spatiotemporal localisation of dying cells. Yet, despite fast progress in our understanding of the molecular regulators of programmed cell death and extrusion, we still are unable to predict when, where and how many cells will die in a tissue. This most likely relies on multiple feedback mechanisms that can modulate death rate/cell extrusion at various spatial and temporal scales (Villars and Levayer, 2022), including activation of the pro-survival signal ERK (also known as MAPK1) in the neighbouring cells (Bock et al., 2021; Gagliardi et al., 2021; Valon et al., 2021), long-range coordination for cell extrusion (Aikin et al., 2020; Takeuchi et al., 2020) as well as positive feedback on apoptosis (Perez-Garijo et al., 2013). Thus, obtaining a comprehensive understanding of epithelial cell death regulation entails the dissection of multi-layered regulations integrating feedback on several spatial and temporal scales as well as several steps of decisions. Such a challenging question requires a highly quantitative dataset on the total number of cell death as well as their precise spatiotemporal distribution and high-throughput methods to compare these values in various perturbed backgrounds.

Recent advances in long-term live imaging have provided a wealth of data regarding tissue dynamics, especially for epithelia in 2D. However, retrieving cellular events quantitatively in a high-throughput manner remains extremely challenging. For instance, more than 1000 extrusions, distributed all over the epithelium in the *Drosophila* pupal notum, have been observed in less than 12 h (Guirao et al., 2015; Valon et al., 2021). So far, quantitative analyses of cell death/extrusion have been performed using laborious manual detection of these events (Moreno et al., 2019; Valon et al., 2021; Villars et al., 2022). This highly time-consuming task remains one of the main bottlenecks for comparing a high number of conditions with precise quantitative readouts. Alternatively, automatic epithelial cell death detection has been performed through systematic segmentation and tracking of all the cells (Etournay et al., 2016; Guirao et al., 2015). However, this method typically entails extensive manual corrections even when using machine learning-enhanced segmentation (Aigouy et al., 2020), and still represents an important load of work for large fields of view and long timescales, hindering large-scale analysis on many tissues.

Altogether, these challenges and needs call for an automatic tool that would allow accurate spatiotemporal detection of cellular events without relying on systematic segmentation and tracking of cells. Recent progress in computer vision has opened the possibility of automating the detection of objects and patterns in biological images and image series (Hallou et al., 2021). In particular, deep-learning approaches have been used successfully to recognise cellular events, such as cell division and cell death in yeast (Aspert et al., 2022) or in mammalian cell culture (Kabir et al., 2022; La Greca et al., 2021; Mahecic et al., 2022; Phan et al., 2019; Shkolyar et al., 2015). However, this was mostly applied to transmission light microscopy and these pipelines are not applicable to large samples and embryos, where imaging mostly relies on fluorescent and confocal microscopy. As such, there is currently, to our knowledge, no implemented solution for the automatic detection of cell death and cell extrusion events from epithelia *in vivo*.

To answer these challenges, we devised a supervised machine-learning pipeline called DeXtrusion. Detection of extrusion is performed by screening the entire movie with sliding windows and detecting cellular events on each window. The core of our pipeline is based on a recurrent neural network that classifies each image sequence (called DeXNet). These local classifications are then post-processed together at the movie level to convert them to probability maps of the presence of a cellular event and, eventually, precise single-point event detection. We devised and applied this method on the *Drosophila* pupal notum, a single-layer epithelium, using cell contour-labelled epithelia with tagged E-cadherin (Shotgun in *Drosophila*). The method is flexible enough to provide accurate and precise predictions with movies of different temporal resolutions, pixel sizes and imaging set-ups as well as different E-cadherin labelling, without any need for segmentation. Moreover, DeXtrusion generalises well in the context of other epithelia (e.g. pupal abdomen, pupal wing), which can be further enhanced by re-training on small data sets. The same methodology can also detect other cellular events, such as cell division and cell differentiation. By resolving the bottleneck of extrusion/cell death detection, DeXtrusion will open the way for a more systematic and quantitative characterisation of cell death distribution in large datasets, which will be essential to understand its multi-layered regulation. The same methodology could easily be applied to any cellular event detected by fluorescent live imaging. DeXtrusion is distributed as an open-source Python module on GitLab (https://gitlab.pasteur.fr/gletort/dextrusion) along with our trained neural networks and scripts (Jupyter notebooks and Fiji macros) to facilitate its usage; our annotated datasets used for training of the pipeline are available on Zenodo (Villars et al., 2023) (https://doi.org/10.5281/zenodo.7586394).

## RESULTS

### DeXNet: a neural network to recognise extrusion in cropped image sequences

**Fig. 1.**
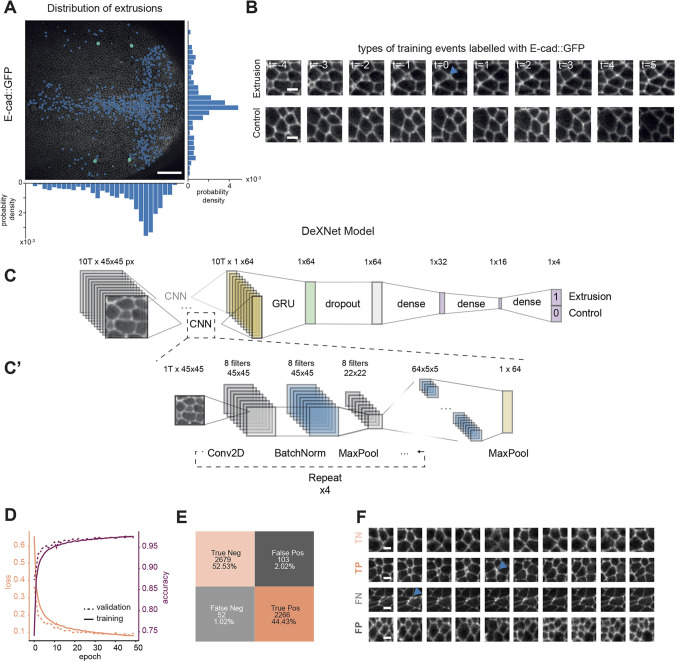
**DeXNet, a neural network to recognise cell extrusions in image sequences in epithelia.** (A) Snapshot of a developing *Drosophila* pupal notum marked with E-cad::GFP (knock-in) with overlaying extrusions manually detected over 16 h of development. Histograms represent the distribution of cell extrusions along the anteroposterior axis (bottom) and along the left-right axis (right) for a total of 720 extrusions. Scale bar: 50 µm. (B) Representative image sequence centred on an extrusion event (top row, blue arrowhead points at the end of an extrusion), or representing a control sequence with no event (bottom row). The time relative to the extrusion event is given in each frame (one frame=5 min). Scale bars: 5 µm. (C,C′) Schematic of the architecture of the DeXNet model. (C) Main model. The image sequence, composed of ten time frames (T) of 45×45 pixels (px), is passed first to a convolutional neural network (CNN; detailed in C′), which encodes each image of the sequence in a vector of 64 features. The resulting matrix for the sequence (10T×64, representing ten time points) is itself fed into a gated recurrent unit (GRU) to consider temporal information. This is then passed to a dropout normalisation layer before going through a sequence of densely connected layers (‘dense’). Finally, it predicts with a probability whether the input sequence is an extrusion or not. (C′) Detailed architecture of the encoding CNN. Each image in the sequence goes into a sequence of convolution (Con2D), batch normalisation (BatchNorm) and max pooling (MaxPool), which is all repeated four times. The convolution was made using *n*=8 filters, which were multiplied by two at each layer, thereby increasing the first dimension (8,16,32,64) whereas the max pooling reduces the other dimension (45,22,11,5). Finally, this output (64×5×5) goes through a final step of max pooling encoding the image in a final vector of dimension (1×64). (D) Loss (left *y*-axis) and accuracy (right *y*-axis) curves for training data (solid lines) and validation data (dashed lines). The loss function is an estimation of the distance between the prediction and the real data (equivalent to an error estimation). Accuracy is defined by the proportion of correct predictions out of all the predictions (see Materials and Methods). (E) Confusion matrix showing the accuracy of the DeXtrusion model. Orange boxes show the number of correctly predicted events (light orange are true negatives, dark orange shows true positives). Grey events are the number of wrongly predicted events (light grey are false negatives and dark grey show false positives). (F) Representative image sequences showing example events for each of the categories shown in E (FN, false negatives, i.e. extrusions wrongly classified as control events; FP, false positives, i.e. control events wrongly classified as extrusions; TN, true negatives, i.e. events correctly classified as controls; TP, True positives, i.e. extrusions events correctly classified as extrusions). Blue arrowheads indicate the end of extrusion events. Scale bars: 5 µm.

We first aimed to detect cell extrusion in the *Drosophila* pupal notum: a single-layer epithelium on the back of the developing *Drosophila* pupal thorax with a high rate of cell extrusion/apoptosis that follows stereotypical patterns (Fig. 1A) (Guirao et al., 2015; Levayer et al., 2016; Marinari et al., 2012; Valon et al., 2021; Villars et al., 2022). We used a dataset generated in the laboratory covering a large number of extrusion events (6700 in the training set and 2320 in the test set; Tables S1 and S2). This dataset was made of large-scale movies of the pupal notum obtained from two different imaging set-ups (see Materials and Methods), with different frame rates and signal-to-noise ratios, and different fluorescent proteins coupled to the adherens junction protein E-cadherin. It contains wild-type (WT), mutant and drug-perturbed conditions to obtain a model robust in a large range of conditions (Tables S1 and S2). Cell extrusions were manually annotated by clicking on the point of termination of apical constriction whereas control positions were drawn randomly, excluding locations containing an extrusion (see Materials and Methods for details regarding event detection). To handle data with different frame rates and spatial resolutions, we fixed a reference spatiotemporal scale (0.275 microns/pixel and 5 min/frame) on which the neural network was trained. Every processed movie was rescaled to this reference before being processed.

**Fig. 2.**
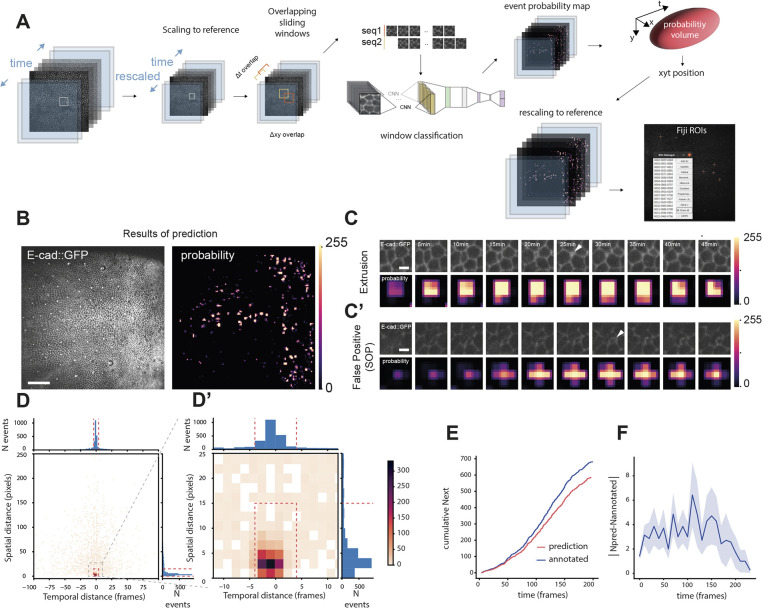
**DeXtrusion pipeline to detect extrusions on full movies.** (A) Schematic of the DeXtrusion pipeline. The input movie is first rescaled to the reference scale. Then, cropped image sequences are extracted, spanning the entire movie with an overlap both spatially and temporally. Each image sequence is then processed through our neural network DeXNet. The resulting probability of the presence of an extrusion is added around the central position of the image sequence to build a probability map on the entire movie, before rescaling it to the original movie size. Single-point events can also be generated by taking the centroids of high probability volumes in the probability map and exporting them as a Fiji ROI file. (B) Projection of a resulting probability map. Snapshot of the input movie, an epithelium labelled with E-cadherin-GFP (left) with the corresponding probability map (right). Probabilities are drawn as a colour map, with values converted to a 0 (black) to 255 (white) scale for visualisation. Scale bar: 50 µm. (C,C′) Example of a correctly detected extrusion (C) and a false-positive detection (C′) (here a sensory organ precursor, SOP). Top rows: Image sequences of E-cadherin::GFP (top) from the original movie cropped around a correctly (C) or wrongly (C′) detected extrusion event. Bottom rows: The corresponding extrusion probability. Probabilities are colour coded from 0 (black) to 255 (white). White arrowheads indicate detected events. Scale bars: 5 µm. (D,D′) 2D histogram of the spatiotemporal distances between manually annotated extrusions and DeXtrusion results on the seven test movies. The colour code represents the number of extrusions detected within a given temporal distance (*x*-axis, in time frames; 1 frame=5 min) and a given spatial distance (*y*-axis, in pixels; 1 pixel=0.275 µm). The red dashed rectangle represents the events that are considered as matching with a manually annotated event (below the spatial and temporal threshold; see Materials and Methods). The histogram of distribution for spatial distance is shown on the right, and the one for temporal distance is on the top. The thresholds are represented by red dashed lines. (D′) Close-up view of the histograms of spatial and temporal distances for the area containing the most observations (see grey square in D). (E) Cumulative number of extrusions during developmental time for DeXtrusion results (red) and manually annotated results (blue). Extrusions were detected on one test movie of 942×942 pixels and 200 frames. (F) Average absolute difference of the number of extrusions over time between DeXtrusion detections and manually annotated extrusions. Shaded area represents s.e.m. Data taken from all test set movies (binned every 10 min from the start of the movie).

To detect with spatiotemporal accuracy all cell extrusions, we generated a pipeline called DeXtrusion, which screens through the entire movie using overlapping sliding windows and assigns a probability of extrusion for each cropped sequence. DeXtrusion refers to the full pipeline detecting cellular events from the full movies (see below and Fig. 2), whereas DeXNet refers to the neural network used to classify each cropped image sequence. Each cropped image sequence (a series of ten images, one every 5 min, of 45×45 pixels in the reference scale, representing two or three cells per frame) was processed through a neural network called DeXNet to estimate the presence or absence of an extrusion event (Fig. 1B). For this, we built and trained a neural network to classify each image sequence (Fig. 1C,C′). We first chose an architecture based on a convolutional neural network (CNN), an efficient approach for image-based classification (LeCun et al., 2015). Because cell extrusion is mostly defined by the dynamics of cell shape changes (progressive apical constriction), we decided to include temporal information by using a recurrent neural network architecture. We chose a gated recurrent unit (GRU) architecture, which computes and propagates temporal information while preserving a parsimonious network architecture (Cho et al., 2014). Each image of the temporal sequence is first encoded using the CNN (Fig. 1C′) and reduced in a vector of discriminant features (64), which is then combined with the other temporal image feature vectors into the GRU (Fig. 1C). The output of the time-distributed GRU layer then goes through a sequence of densely connected layers that perform the final classification (Fig. 1C, purple). Eventually, the network provides a probability of containing an extrusion for each cropped image sequence. This probability is finally thresholded to a binary output (extrusion or control event). We trained DeXNet on image sequences extracted from our dataset, split in a training and test set (25 versus seven movies; see Materials and Methods). We took cropped image sequences in the reference scale centred spatially and temporally around the manually detected extrusions (time 0 being defined by the termination of apical constriction; Fig. 1B), whereas control sequences were made of similar cropped image sequences that did not contain any event (see Materials and Methods). To avoid imbalanced detection in our training dataset (due to the over-representation of ‘no event’ sequences relative to extrusion), we equalised the number of extrusion and control sequences used for the training (see Materials and Methods). The size of the temporal window, the *xy* size of the cropped input images, as well as other hyperparameters of the model [e.g. the number of epochs (the number of times the dataset is processed by the network for training), the number of features, data augmentation], were tuned and optimised by testing several sets of values (Figs S1-S3). Parameters were compared after processing through the full DeXtrusion pipeline (see below and Fig. 2) by comparing the precision (the number of true positives out of all detected events), the recall (the proportion of manually annotated cellular events detected by the pipeline), the computation time, and the F1 score (a measurement of quality and exhaustivity of detections; see Materials and Methods). For instance, the temporal size of the sequences (ten frames, 50 min) was chosen such that it contains enough information to recognise extrusion (which lasts on average 20-30 min; Villars et al., 2022) while reducing the chance of capturing two events and reducing calculation time (see Materials and Methods; Fig. S1A-D). Importantly, we observed a drastic reduction of precision and recall when restricting the time windows to two frames, illustrating the key role of temporal information for accurate detection (Fig. S1A-D). Taken together, the selected hyperparameters allowed the model to converge properly under 50 epochs and took around 55 min of training with 1 GPU (Fig. 1D). Compared with our manual annotation, the classification of the neural network on cropped image sequences extracted from our test dataset resulted in an accuracy of 97% (the proportion of correct predictions out of all predictions; see Materials and Methods), with 2% false positives (detected extrusions that were not real extrusions) and 1% false negatives (real extrusions that were not detected) (Fig. 1E,F). Of note, these 2% of false positives could constitute a challenge for detection in the full movies because the proportion of ‘no event’ sequences is much higher than the proportion of real extrusions. This may generate a significant proportion of false positives (thus reducing our ‘precision’). We will discuss the optimisation of the precision in later sections.

### DeXtrusion: from cropped images classification to whole-movie extrusion detection

Thus far, we have demonstrated that our trained neural network DeXNet provides a fast and efficient classification of each image sequence. To detect extrusion events across the tissue and at all temporal time points, the complete movie was split into 45×45 pixels cropped images with 50% overlap. The time axis was divided into sequences of ten frames every two time points after rescaling the time to the reference scale (Fig. 2A). These parameters are a good compromise between the spatiotemporal precision of the results and the speed of computation and can be tuned by users. For each sliding window, DeXNet provides a single probability of extrusion. This value is then allocated in the original movie in a 22×22 pixels and five time-point regions around the centre of the cropped sequence. The final local probability output is then obtained by averaging for each pixel the probability of the different overlapping windows (in time), generating an extrusion probability map of the whole movie with a spatial resolution of 22 pixels in the reference scale (Fig. 2A,B, Movie 1). This output, once rescaled back to the spatiotemporal scale of the original movie, can be directly used to screen visually the detection of extrusion events and assess putative tissue patterns (Fig. 2B).

To count the number of and precisely localise cell extrusion events, this probability map must be converted to single-point event detection. Single events in our probability map are associated with high probability within several consecutive time windows and on a given *xy* surface, thus generating a volume (*xyt*) of pixels with high probability (Fig. 2A,C, Movie 1). We first thresholded the probability map to obtain a 3D mask of positive detections. To filter out false positives, which are usually detected on smaller areas and fewer time windows (Fig. 2C,C′), we also thresholded the size of the positive volume to fit the minimal volume associated with extrusion events (which last between 20 and 30 min and should cover at least one cell diameter; Villars et al., 2022). The probability threshold and volume threshold were optimised to maximise the precision and recall (Fig. S2, Materials and Methods). We also used a watershed separation to separate close events. Eventually, the exact location of the extrusion is defined by the centroid of the high-probability volume. Detected extrusions are then exported as a list of point regions of interest (ROIs) compatible with Fiji ROI manager (Schindelin et al., 2012) (Fig. 2A).

To assess the performance of DeXtrusion, we compared the resulting ROIs with the manually annotated ROIs of our test dataset. We first measured systematically the *xy* Euclidean distance and time distance between manually annotated and automatically detected extrusions (Fig. 2D,D′). This showed that 70.6% of the detections were below a 15-pixel distance (4.12 µm or approximately one cell radius) and within (±) four time frames (±20 min). We then classified detections as correct for spatial distance below 15 pixels and within four time frames between the DeXtrusion and manually located ROIs. Doing so, we obtained a recall of 0.87 (the proportion of manually annotated cellular events detected by the pipeline) and a precision of 0.46 (the proportion of detected events that are indeed extrusion, averaged on five independent trained networks). We will discuss the optimisation of the precision in the next section. Finally, we measured systematically the total number of detected extrusions compared with manually annotated events at every developmental time point to check whether the accuracy of our detection is sensitive to the developmental stage. We observed a fairly constant error (around three errors every ten frames/50 min), suggesting that our methodology is robust at all developmental stages imaged in the notum (Fig. 2E,F). Thus, our methodology can retrieve the vast majority of extrusion events at any stage of development.

### Optimisation of the model for the detection of extrusion

**Fig. 3.**
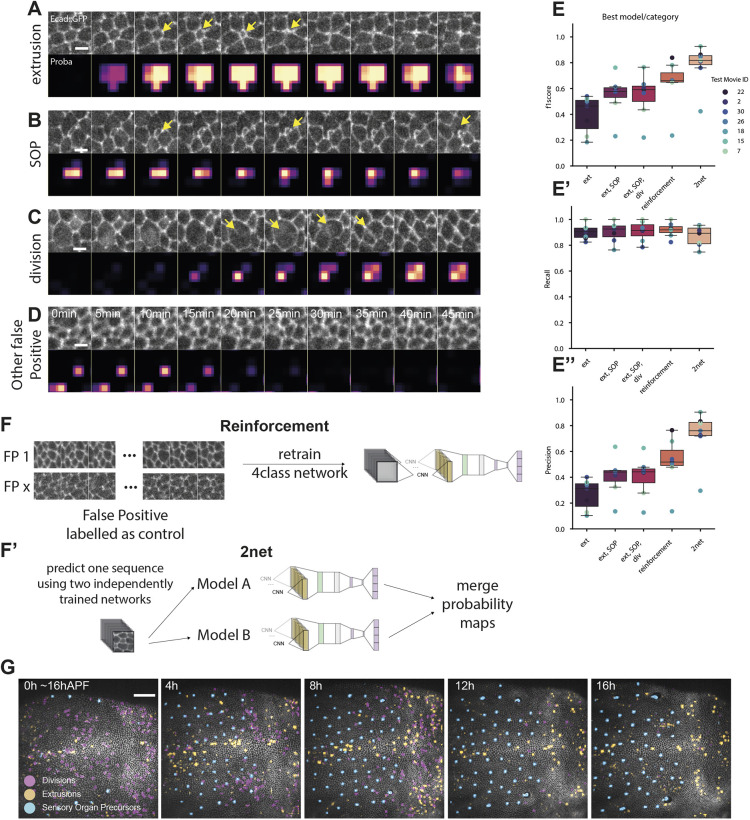
**Optimisation of the model for the detection of extrusions.** (A-D) Image sequence events correctly predicted as extrusions (A) or not (B-D) by the initial DeXnet trained on two classes only (extrusion or no event). Top rows of each panel show the image sequence, bottom rows show the probability maps obtained by DeXtrusion. Scale bars: 5 µm. (A) Image sequence representing an extrusion and its associated probability map. Yellow arrows point to the apical closure of the extruding cell. (B) Image sequence representing a forming sensory organ precursor (SOP) wrongly predicted as an extrusion and its associated probability map. Yellow arrows point to the small cell of the SOP, which constricts and leads to misclassification. (C) Image sequence representing a dividing cell wrongly predicted as an extrusion and its associated probability map. Yellow arrows point to the furrow formation during cytokinesis. (D) Image sequence representing a control event wrongly predicted as an extrusion and its associated probability map. (E-E″) Changes in the model to optimise its prediction scores on extrusion for F1 score (E), recall (E′) and precision (E″), with the initial two-class model (ext: extrusions and no event); the inclusion of SOPs (ext, SOP: extrusions, SOPs and no event); the inclusion of SOPs and cell divisions (ext, SOP, div: extrusions, SOPs, cell division and no event); including the three cellular events and reinforcement (reinforcement; see below); and using two independent networks (2net; see below). The results are shown for the best-trained network for each class model (see Fig. S2G-I for the averaged data). (F,F′) Schematics of the steps added to the four classes model to increase its F1 score on predicting extrusions. Box plots show the median, the first and third quartile. Top and bottom bars are the maximal and minimal values. (F) Reinforcement consists of taking regions covering no event misclassified as extrusion, cropping them and adding them to the training set with a control label. This forces the model to learn that the previously misclassified events are in fact controls (no event). (F′) 2net uses the two best independently trained models (stochasticity results in models with slightly different weights and biases) and averages the probability map from these two models. (G) Image sequence showing the results of DeXtrusion predictions after optimisation for extrusions (orange), cell divisions (pink) and differentiation (SOPs, blue) on the full-scale movie (pupal notum, local projection of E-cad::GFP) and over time. Time is shown in hours after pupal formation (hAPF). Scale bar: 50 µm.

To this point, we had designed and optimised DeXtrusion to detect exhaustively all the extrusion events, which led to a high recall of 0.87 (87% of events detected). However, we had not yet fully optimised the methodology to filter out detection of false positives (to optimise the so-called precision). Indeed, many cellular events which share some phenotypic similarities with extrusion are very frequently mislabelled as extrusion (Fig. 3A-D, Movie 1). This includes, for instance, the formation of sensory organ precursors (SOPs), which, through asymmetric cell division, form cells with very small apical areas (Gho et al., 1999) that are often detected as extrusions (Fig. 3B). Similarly, the shrinkage of cell apical area concomitant with cytokinesis and furrow formation are frequently misclassified as extrusions (Fig. 3C). Thus, to enhance the precision of DeXtrusion (the proportion of correct extrusion detection), we decided to add these other cellular events in DeXNet to discriminate them from extrusion. We trained the network to detect cell extrusions, cell divisions and SOPs, or the absence of events, using manually annotated events in our dataset (6700 extrusions, 3021 divisions and 3054 SOPs; Fig. 3F). DeXNet was able to categorise the cropped image sequences into these four classes with high accuracy in our training set (0.986, 0.989 and 0.976 for, respectively, extrusions, SOPs and divisions; Fig. 3G, Movie 1). By including this new DeXNet in our pipeline, we significantly increased the precision of DeXtrusion (from 0.26 to 0.41, for a single run with the selected networks having the highest recall; Fig. 3E,E″), but this did not impact significantly the recall (Fig. 3E′). This is also reflected by the F1 score (Fig. 3E), a measurement of the quality and exhaustivity of detections (see Materials and Methods), which increased from 0.40 to 0.54. To enhance our precision further, we then manually screened all remaining false-positive detections and included image sequences representative of the patterns of these false positives in the training inputs as control sequences and re-trained the DeXNet network (using the network including the four classes of events). This reinforcement increased the model's precision to 0.52 for similar recall (0.92 compared with 0.90). Finally, we exploited the inherent stochasticity of the training process (which always leads to different network weights and biases) to confront the predictions from two independently trained networks (Segebarth et al., 2020). Averaging these two independent classifications had the most significant effect and increased the precision to 0.72 (recall of 0.86 and F1 score of 0.78). This barely affected the recall (Fig. 3E-E″, 2net), but it increased the calculation time by a factor of two. Of note, the accuracy was much lower for the test movie of the colcemid-injected pupae (Fig. 3E-E″, movie ID 18), the phenotype of which was poorly represented in the training dataset and corresponded to an extreme condition in which the mode of extrusion and the architecture of the tissue are different (Villars et al., 2022). After all these optimisation steps, the final model (2net) could still detect all cellular events in a movie covering the full pupal notum (1200×1200 pixels, 200 time points) in less than 40 min on a regular PC with 1 GPU. Taken together, these optimisations steps led to a drastic increase in precision while maintaining similar recall on test movies. The last remaining false positives (∼20%) can be easily filtered out manually from the final ROIs list. We estimated that the full procedure (DeXtrusion computation time+manual correction) takes 1-2 h for a movie covering the full pupal notum over 200 times (representing roughly 1000 extrusions). This corresponds to a drastic time saving compared with the exhaustive manual detection of extrusions [∼10 h for a trained user (Valon et al., 2021) versus approximately 1 h of manual correction with DeXtrusion].

### Generalisation of DeXtrusion to mutant contexts and other epithelia

**Fig. 4.**
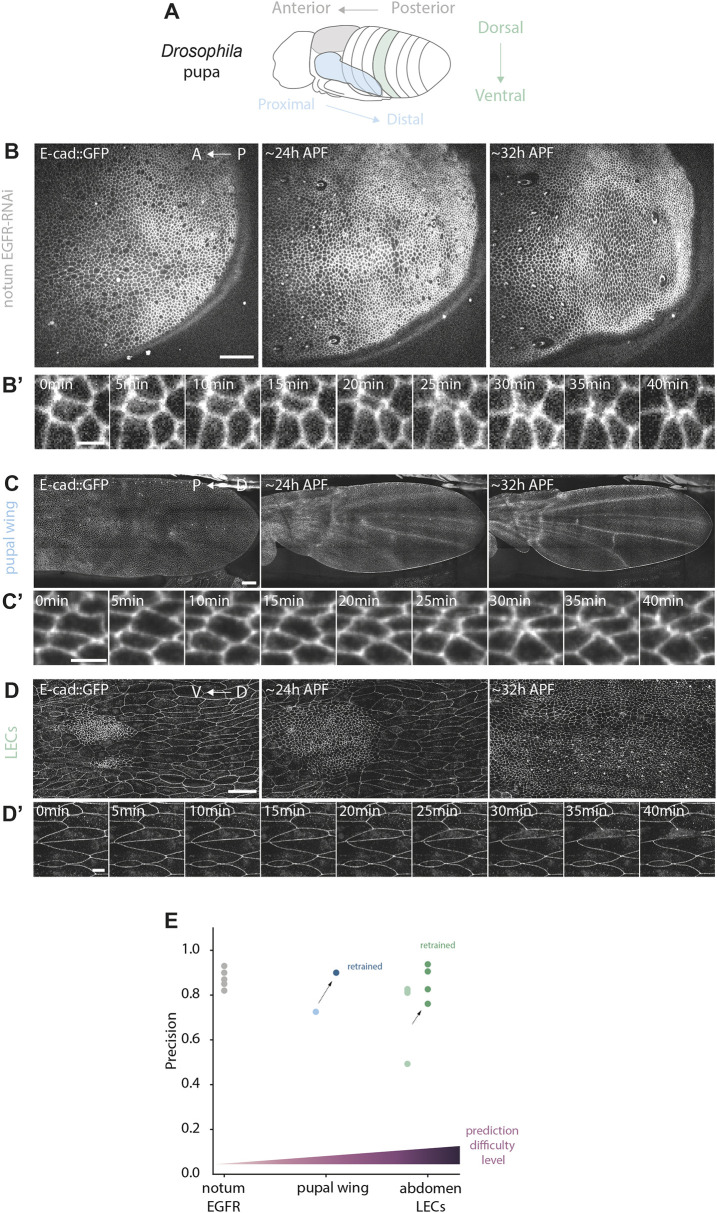
**Generalisation of DeXtrusion to the mutant context and other epithelia.** (A) Schematic of a *Drosophila* pupa highlighting the different epithelia on which the model was tested. The model was trained on the notum (grey) and tested on the pupal wing (blue) and finally on the abdominal larval epithelial cells (LECs, green). (B-D′) Example image sequences of the different tissues used to test the generalisation of DeXtrusion and the corresponding extrusion phenotypes. (B) Pupal notum epithelium expressing an RNAi against EGFR. Scale bar: 50 µm. (B′) Example image sequence showing an extrusion cropped from B. Scale bar: 5 µm (C) Pupal wing epithelium from Etournay et al. (2015). Scale bar: 50 µm. (C′) Example image sequence showing an extrusion from C. Scale bar: 5 µm. (D) Pupal abdomen epithelium extracted from Davis et al. (2022). Results were computed only for LECs (bigger cells), excluding the histoblasts (smaller cells). Scale bar: 50 µm. (D′) Example image sequence showing an extruding LEC from D. Scale bar: 10 µm. (E) Manually computed precision for the prediction on different tissues with increasing differences compared with tissues for which DeXtrusion had been trained. Gray shows the precision of DeXtrusion for notum expressing an RNAi against EGFR (example shown in B,B′). Light blue is the precision of DeXtrusion for the pupal wing epithelium (example shown in C,C′). Dark blue is the precision of DeXtrusion on the same tissue after re-training the model on a subset of extrusions. Light green is the precision of DeXtrusion on LECs (example shown in D,D′). Purple is the prediction on the same tissue after re-training. Darker purple is the prediction on the same tissue after re-training and reinforcement. The raw values of precision and recall can be found in Table S4. A, anterior; D, dorsal; P, posterior; V, ventral.

The performance of a neural network is usually highly limited to the training data range and cannot easily generalise to other conditions/tissues not represented in the training dataset (Mockl et al., 2020). To test the capacity of generalisation of DeXtrusion, we challenged our pipeline by testing its performance on different data from relatively similar biological conditions as well as very different biological contexts. We first trained two ‘full’ networks, using all our annotated data (training+test dataset). To adapt DeXtrusion to other tissues (Fig. 4A) in which the cell size and duration of cellular events are not of the same scale, the reference scale at which movies are resized is calculated such that the cell diameter is around 25 pixels with extrusion taking place over four or five frames. We first tested DeXtrusion on *Drosophila* pupal notum depleted for EGFR (UAS-EGFR-dsRNA driven by pnr-Gal4), a condition that modifies tissue shape and the spatiotemporal distribution of extrusion with minimal effect on the extrusion process per se (Moreno et al., 2019; Valon et al., 2021). We obtained an overall good detection level in this context (Fig. 4A-B′,E, Movie 2). Because annotated data do not exist for these movies, we randomly sampled the prediction results to check the events manually (see Materials and Methods). Using this method, we estimated that 87% of extrusion detections were correct (precision=0.87; Fig. 4E). We further challenged DeXtrusion using the *Drosophila* pupal wing, an epithelial tissue with similar cell shape and size to the pupal notum (Aigouy et al., 2010; Etournay et al., 2015; Farhadifar et al., 2007) (Fig. 4A,C,C′, Movie 3). Using a previously published movie of E-cad::GFP pupal wing (Etournay et al., 2015), we obtained a precision of 0.786 (excluding ROIs outside the wing; see Materials and Methods; Fig. 4E) and an estimated recall of 0.85 (based on 59 extrusions manually annotated). These scores were significantly improved by re-training DeXNet on a small proportion of manually annotated extrusions and divisions from the pupal wing (75 events), reaching a precision of 0.91 and an estimated recall of 0.83 (Fig. 4E, Movie 3; see Materials and Methods).

We further challenged DeXtrusion by testing its performance on a squamous epithelial tissue, the larval epithelial cells of the pupal abdomen, where cells have very different shapes from the pupal notum and where extrusions occur through slightly different mechanisms (Hoshika et al., 2020; Michel and Dahmann, 2020; Teng et al., 2017; Villars et al., 2022) (Fig. 4A,D,D′). This test was conducted on four movies taken from previous publications (Davis et al., 2022; Tapon and Salbreux, 2022) focusing exclusively on the larval epidermal cells. After proper rescaling, we obtained a precision of 0.737 but a low recall of 0.38 (using available segmentation and annotation; Davis et al., 2022; Tapon and Salbreux, 2022), despite adjusting the threshold distances (spatial and temporal) to the scale of the movie (see Materials and Methods). This may reflect the difference in cell morphology (long and curved junctions) and dynamics of extrusion (progressive loss of E-cad and rounding; Teng et al., 2017) (Fig. 4D′). To adapt DeXtrusion to these cells, we re-trained our DeXNet models using the annotated extrusions of one of the four movies. The precision increased to 0.857 and the recall nearly doubled to 0.69 (Fig. 4E, Movie 4). Increasing the re-training data to two movies continued to improve the performance, but less drastically (precision of 0.86 and recall of 0.74).

Altogether, these data demonstrate that DeXtrusion can robustly detect extrusion events on various tissues and conditions. For situations in which cell shape and the profile of extrusions are very different, some additional training is sufficient to reach back good precision and recall, illustrating the adaptability of DeXtrusion.

## DISCUSSION

DeXtrusion allows fast and precise detection of cell extrusions in a wide range of imaging conditions without any need for a segmented dataset and can also detect accurately other cellular events (SOPs, cell divisions). Although our precision (∼0.8) still requires a manual correction phase to filter out false positives, this leads to a considerable gain of time compared with the manual annotation of large movies (from 10 h to 1 h). This opens new opportunities for systematic quantification of the spatiotemporal distribution of cell death in a large number of movies. DeXtrusion offers the possibility of screening for drugs or mutations affecting the spatiotemporal distribution of cell death and could lead to the identification of new biological factors modulating cell apoptosis and new spatiotemporal feedback mechanisms. We extended DeXtrusion to detect cell divisions and SOPs with the aim of improving our precision, but these additional features could also be useful for analysis of the spatiotemporal interplay between these three cellular events, for instance the coupling between cell death and cell division and compensatory proliferation (Fan and Bergmann, 2008; Kawaue et al., 2021 preprint; Mesa et al., 2018). Combining such large datasets with graphical neural networks (Yamamoto et al., 2022) and spatiotemporal point pattern analysis and modelling (Valon et al., 2021) may eventually help to rapidly detect new spatiotemporal couplings at various *xyt* distances. Note, however, that we did not estimate the precision or recall of our pipeline in detection of these other cellular events as it was not the initial scope of our study.

We challenged DeXtrusion to detect extrusions in other tissues and/or new genetic backgrounds and demonstrated how easily it could be adjusted using, when necessary, minimal re-training datasets (by manually detecting a few events and without the need for segmentation). This also included epithelia with very different architectures (cuboidal in the notum, squamous in larval epidermal cells). We have not tested the performance of DeXtrusion on tissue with different labelling methods (e.g. membrane, actin), but reasonable re-training may be enough to readjust the pipeline for a wide range of tagged proteins. Alternatively, integrating purely geometrical features in the training dataset (through segmentation of a large number of extrusions/divisions/SOPs and integrating skeletonised sequences in the training) may help to build a more generalised model that is less sensitive to the tagged protein and the variation of fluorescence intensity. Although collecting enough segmented data may be time consuming, it could be a very promising alternative method for building a very generalised model. Introducing manually defined features into the neural network could also be an interesting approach to increase the network performance (Huang et al., 2022), but this would require more computation to extract the hand-crafted features. Although we have limited our detections to extrusions, divisions and SOPs, the same pipeline could easily be trained to recognise new cellular events. Of note, the detection of cell extrusion is particularly challenging because the deformations associated with cell extrusion are not stereotypical: some cells constrict isotropically and form clear rosettes, whereas others constrict while elongating and progressively losing cell–cell junctions (Levayer et al., 2016; Marinari et al., 2012; Villars et al., 2022), as opposed to, for instance, cell divisions, which are always preceded by cell rounding. We are therefore confident that our methodology could be very performant on a wide range of epithelial events. For instance, a significant proportion of false positives were related to local cell rearrangements and T1 transitions (Guirao and Bellaiche, 2017). Adding this new feature could not only increase the precision of our pipeline, but also offer an interesting tool for large-scale analysis of tissue dynamics and the evolution of fluidity in time and space (Tetley et al., 2019). Interestingly, Gallusser et al. (2023 preprint) proposed recently a self-supervised pipeline to capture automatically the presence of an event based on its asymmetric signature in time. Filtering the movie to keep only potential events with their framework before performing the event classification through DeXNet could be an additional boost to our pipeline.

The workflow that we have developed here for extrusion detections could be applied to any other biological event detection, provided that enough training data are available, without the need for segmentation. Moreover, the high rate of false positives of our initial pipeline revealed the difficulty of detecting one particular type of infrequent event in a movie containing potentially other events. The strategies we applied to improve the precision (classifying other cellular events as well, reinforcement of the training by including other typical false positives, e.g. transient cell deformations, and averaging the prediction of several networks) led to a drastic increase in the prediction precision (approximately threefold increase). To our knowledge, these methods were not commonly used previously in a deep-learning context and could, in principle, be applied to any other machine-learning pipeline. By distributing DeXtrusion as an open-source resource, we offer an optimised pipeline that can be easily tuned to detect biological events in temporal series without segmentation and tracking.

## MATERIALS AND METHODS

### Generation of the training dataset

To create an annotated dataset, we used movies that had been previously manually annotated for extrusions in the laboratory for other studies (Moreno et al., 2019; Valon et al., 2021; Villars et al., 2022). Pupae were dissected and imaged on a confocal spinning disc microscope (Gataca systems) with a 40× oil objective (Nikon plan fluor, N.A. 1.30) or 100× oil objective (Nikon plan fluor A N.A. 1.30) or an LSM880 equipped with a fast Airyscan using an oil 40× objective (N.A. 1.3), *z*-stacks (1 μm/slice). All the movies were built using a local *z*-projection plugin that follows tissue curvature (Herbert et al., 2021). We pooled 32 movies, all from the *Drosophila* pupal notum, but with different acquisition setups, genetic backgrounds and junctions staining. The full list of movies and their characteristics is given in Tables S1 and S2. We split the annotated data into two datasets, one for training (Table S1) and a smaller one for testing (Table S2). For this, we selected randomly one movie of each of our different conditions for the test set, and all remaining movies of the same condition were used for training. We obtained two datasets: the training set of 25 movies, with 6692 annotated extrusion events from eight different conditions, and a test set of seven movies, with 2320 annotated extrusions from seven different conditions. These data with the manual annotation are freely available in a repository (https://doi.org/10.5281/zenodo.7586394) (Villars et al., 2023). Detection of cellular events (extrusion, SOPs, cell division and control regions) were performed manually on Fiji using dot ROIs and extracting *xyt* coordinates. For extrusion, the point of termination of apical constriction was manually selected. For cell division, the centre of the dividing cell just prior to cytokinesis (beginning of furrow constriction) were selected. For SOPs, no specific time points were picked and all the SOPs stage were randomly selected (from the first asymmetric division events to the late SOPs) by clicking in the centre of the cell with the smallest apical area.

For each network training, the training dataset was split in a 75% and 25% proportion between training and validation data, respectively. Note, however, that these two subsets were not fully independent as they are composed of windows extracted from the same movies (training movies).

### Training image sequence generation

To generate training image sequences from the ROI files and not keep all the movies in the running memory, we implemented a movie generator (MovieGeneratorFromRoi.py). This randomly selects the ROI from the input file (after manual detection of events on Fiji; see above) and saves a cropped image sequence centred around that point, with a small spatial and temporal random shift (so that the event is not perfectly centred in the window to limit a bias toward the centre of the image). For control (no event) windows, positions were randomly drawn in the movie and kept if there was no ROI in it.

### Data augmentation

#### Movie augmentation

Movies acquired at higher spatial and temporal resolution are much smaller compared with the majority of movies once rescaled in the reference scale. As such, this kind of data was under-represented in the training data, with only a few events per movie. To reduce this bias, we doubled these movies by downsampling temporally each original movie two times and introducing a shift in the frames extracted between the two repetitions. These movies are indicated with ‘_aug’ at the end of the names in the available dataset.

#### Image-sequence augmentation

We also performed data augmentation on the cropped image sequences used for training. To augment the generalisation of the training, augmentation was performed on each training window with the addition of small temporal and spatial shifts, Gaussian noise, white/black squared, and illumination on all windows or one time frame.

### Data imbalance

The events that we classified (nothing, extrusion, division, SOP) do not have the same frequency in all movies. To reduce the possible imbalance between the representation of these events in the training data, we reduced the number of windows of each over-represented event used in the training such that the number of these events in the training data was similar to the number of less-represented events. This option can be turned off in the pipeline with the Boolean parameter ‘balance’.

### Data rescaling

To homogenise the training dataset and have events of similar duration/size, we rescaled all our dataset to the same temporal and spatial resolution of 5 min/frame and 0.275 µm/pixel. Rescaling of the movies and ROIs was carried out with a Fiji macro. DeXNet networks were trained with input windows of this reference scale. A division event was visible on two or three time-frames, cell extrusion on four or five frames, and a cell had a typical diameter of 25 pixels. Therefore, the DeXtrusion pipeline must rescale all the movies to this reference scale before applying the classification. Because characteristic event durations and cell sizes can vary between tissues/organisms, we used these ‘cellular’ features for rescaling rather than absolute times or distances.

### Data to test generalisation

We tested the robustness of DeXtrusion to different unseen datasets. The first dataset was composed of five movies of *Drosophila* pupal notum depleted for EGFR (UAS-EGFR-dsRNA) (Valon et al., 2021). We do not have manual annotations on these movies, so they were not used in the training data. However, three movies in the original dataset were acquired with the same imaging and genetic conditions. This dataset was thus considered very similar to the training data.

The second dataset was a large movie of the *Drosophila* pupal wing from (Etournay et al., 2015) (3879×1947 pixels, 200 time frames). This tissue was not represented in the training data, but the organisation of the epithelium and cell shape are very similar to those of pupal notum. This dataset was considered similar to training data. We manually annotated a few extrusions (59) spanning the movie to evaluate the recall of DeXtrusion in this sample. We extracted a small part of the movie (495×444 pixels and 200 frames) and annotated this cropped movie to use as re-training data with 28 extrusions, 29 divisions and 19 additional controls for reinforcement. It is important to point out that the re-training window being inside the tested movie does not allow assessment of the performance of the re-training on fully independent data. Moreover, two cell extrusions of the 28 used for re-training were also present in the 59 used for testing, thus slightly biasing the performance assessment. Note that the field of view of the movie contains the whole wing, but also external tissue on the top and bottom parts. We focused the quantification only on the ROIs that were fully inside the wing.

The third dataset was composed of four movies of larval epithelial cells (LECs) of the *Drosophila* pupal abdominal epidermis (Davis et al., 2022). The annotation of cell extrusions in the larval epidermal cells was obtained using the segmentation mask provided in the original dataset (Tapon and Salbreux, 2022) through TissueMiner (Etournay et al., 2016). We focused our test only on larval cells and excluded histoblasts (the nest of small cells) because there is hardly any extrusion in this population and we decided to focus on cells different from the pupal notum. We estimated the typical cell diameter of the larval cells to 80 pixels and the extrusion duration to ten time frames and used these values to rescale the movies for the pipeline. To estimate the recall, we compared DeXtrusion outputs with the generated extrusion annotations on the original movie. However, because cell size and extrusion durations were much higher in these movies, we adjusted the thresholds of spatial and temporal distances to count matching detection (50 pixels *xy* distance, ∼half a cell, and eight time frames). Note that the scores were always calculated on the four movies, even when some were used in the re-training data, which could induce a slight positive bias. However, the two movies used for re-training were the ones on which DeXtrusion results were best even before re-training, and the most drastic improvements came from the two other movies.

### DeXtrusion source code

DeXtrusion is available as an open-source resource on GitLab at https://gitlab.pasteur.fr/gletort/dextrusion under the BSD-3 license. The trained neural networks, Jupyter notebooks and Fiji macros to use DeXtrusion are available and described in this repository. DeXtrusion main code is deployed as a Python module that can be installed through the pip installer package at https://pypi.org/project/dextrusion/. Instructions to install and use DeXtrusion are given in GitLab.

For ease of use, we provide Jupyter notebooks that are dedicated to tasks such as network training, re-training or DeXtrusion detection on new movies. To visualise the results as probability maps or ROIs, Fiji macros are also available. To handle input/output between our Python code and Fiji, we used two specific Python modules in our pipeline: ‘roifile’ (v2022.9.19; https://zenodo.org/record/7094778#.Y-Ik-HbMJdg) and ‘tifffile’ (v2022.5.4; https://zenodo.org/record/6795861#.Y-IkNXbMJdg).

### DeXNet architecture and training

#### Initial training

To categorize sliding windows by taking into account temporal information, we based the architecture of our neural networks on gated recurrent unit (GRU) architecture (Cho et al., 2014). We tested different variations of the architecture and hyperparameters (e.g. number of layers, number of features by layer, number of epochs, size of the sliding windows). The final architecture is represented in Fig. 1C and the full detailed architecture can be found in the source code in the Network.py file. The parameters used for training one network are summarised in the configuration file associated with each DeXNet. For training the neural network to categorise the window as containing an event or not, we used the categorical cross-entropy loss. The number of events classified by DeXNet (control/extrusion, or control/extrusion/SOP or control/extrusion/SOP/division) was managed in the network architecture simply by fixing the size of the final output vector (Fig. 1C, last layer). This final layer is the result of a ‘softmax’ function, so its values (two, three or four values) will add up to 1, like probabilities of event.

In Gitlab, we provide two DeXNet networks trained for control and extrusion classifications (notum_Ext, used for Figs 1 and 2), control, extrusion and SOP classifications (notum_ExtSOP; Fig. 3), control, extrusion, SOP and cell division classifications (notum_EXTSOPDiv; Fig. 3), and trained on all events and all data (train and test) pooled together (notum_all; used for Fig. 4).

#### Optimisation and re-training

The number of categories within the model can be easily tuned by the user and only affects the last layer (densely connected layer) leading to the final prediction. As a result, it is easy to add new classes of cellular events to train or re-train the model and do predictions of these events without affecting the overall architecture of the model. We used that strategy to add the prediction of SOPs and divisions, which were often wrongly labelled as extrusion (false positives) by the pipeline. We then extended that idea by manually selecting events classified as extrusions that were in fact ‘no events’ in the training data. We selected typical false-positive events (e.g. transient cell constriction, loss of focus at the border of the tissue) and forced their classification as ‘no events’ by adding them to the training dataset. Finally, the inherent stochasticity of the training (time sequences generated, weight initialisation, stochastic gradient descent) generated a diversity of final models even when trained on the same dataset. We used that opportunity to optimise the pipeline further by training different models on the same dataset. The two best ones were then combined in the pipeline making independent predictions on the input movies. The output probability maps were then simply averaged, which increased the robustness of the model.

### Evaluation of DeXtrusion results

#### DeXNet evaluation

The performance of DeXNet networks was measured by the accuracy of the results during training, and with the confusion matrix of the classifications on the test dataset after training (Fig. 1E).

#### Pipeline evaluation

##### Comparison with manual annotations

To estimate the quality of DeXtrusion detections, we computed the accuracy [(TP+TN)/(TP+TN+FP+FN)], precision [TP/(TP+FP)] and recall [TP/(TP+FN)] of the resulting ROIs compared with manual annotated ROIs (FN, false negative; FP, false positive; TN, true negative; TP, true positive). ROIs were considered to be the same (between results and manual annotations) if they were within a spatial distance of 15 pixels and a temporal distance of four frames (in the reference scale). The Jupyter notebook dextrusion_CompareRois.ipynb allows us to calculate these scores and to choose the threshold distances to consider ROIs as the same. To consider both precision and recall at once, we also measured the F1 score [TP/(TP+(FP+FN)/2)] to evaluate the performance of our pipeline.

##### Measure of false positives without manual annotations

For movies for which we did not have manual annotations, we could not measure the recall as this would necessitate full annotations of all the events. We estimated the percentage of false positive detection by selecting randomly a high number of resulting ROIs and examining each ROI manually to decide whether the hit was correct or not. The Fiji macro deXtrusion_scoreROIs_Random.ijm allows this and gives the resulting percentage.

##### DeXNet optimisation

Once the model architecture was fixed, we optimised most of the model parameters to achieve the best possible results for the prediction of extrusion. For this, we started with optimisation of the exploration time window (Fig. S1A-D) as it is the first input of the model. We tested different sizes of the time window and kept a size of ten (a good compromise between best score and calculation time). The training gave the best results when the exploration window was temporally centred on extrusion (five frames before, five frames after) (Fig. S1E-G). We then used this parameter to assess the effect of the window's *xy* size. The best results were obtained for a half size of 21 pixels. Although this parameter did not yield the best precision (Fig. S1H), it gave the best recall out of all parameter values explored (Fig. S1I). Recall was the metric we tried to optimise the most to avoid missing any extrusion. Moreover, the prediction time increased linearly with the window size (Fig. S1J). As a result, once pondered by time (precision*recall/prediction time), a size of 21 pixels appears to be the best value (Fig. S1K).

We then explored the impact of the different model thresholds on the model results (Fig. S2). We first fixed a volume threshold of 800 pixels to assess the impact of the probability threshold (Fig. S2A-C). Although the best F1 score was obtained for a probability threshold of 200, it came at the expanse of a lower recall (Fig. S2A,C). Thus, we decided to use a value of 180 for that parameter (second best but higher recall; Fig. S2A) and then explore the impact of the volume threshold following similar reasoning, which led us to pick a value of 800 for that parameter.

Finally, we tried to optimise the hyperparameters (parameters of the model for training) (Fig. S3). First, we explored hyperparameters during the training of a model predicting only two classes (extrusions versus nothing). After parameters exploration, we selected an augmentation value of 3 (Fig. S3A), a number of epochs of 40 (Fig. S3B), a number of initial CNN filters of eight (Fig. S3C) and a number of reinforcements of five (Fig. S3D). We then optimised the model by adding new predicting classes (SOPs and division; Fig. 2) and thus applied the same approach to the model prediction with four classes (extrusions, SOPs, divisions, nothing). Changing the number of epochs had a very limited impact and we therefore kept a number of 40 epochs for the final model and an augmentation value of 2.

All selected parameters are summarised in Table S3.

## Supplementary Material

10.1242/develop.201747_sup1 Supplementary information
