# Supplementary material for: DeXtrusion: automatic recognition of epithelial cell extrusion through machine learning in vivo
Source: Development. 2023 Jun 30;150(13):dev201747. doi: 10.1242/dev.201747 (PMC10323232; doi:10.1242/dev.201747)
Supplement: Supplementary information [file develop-150-201747-s1.pdf]

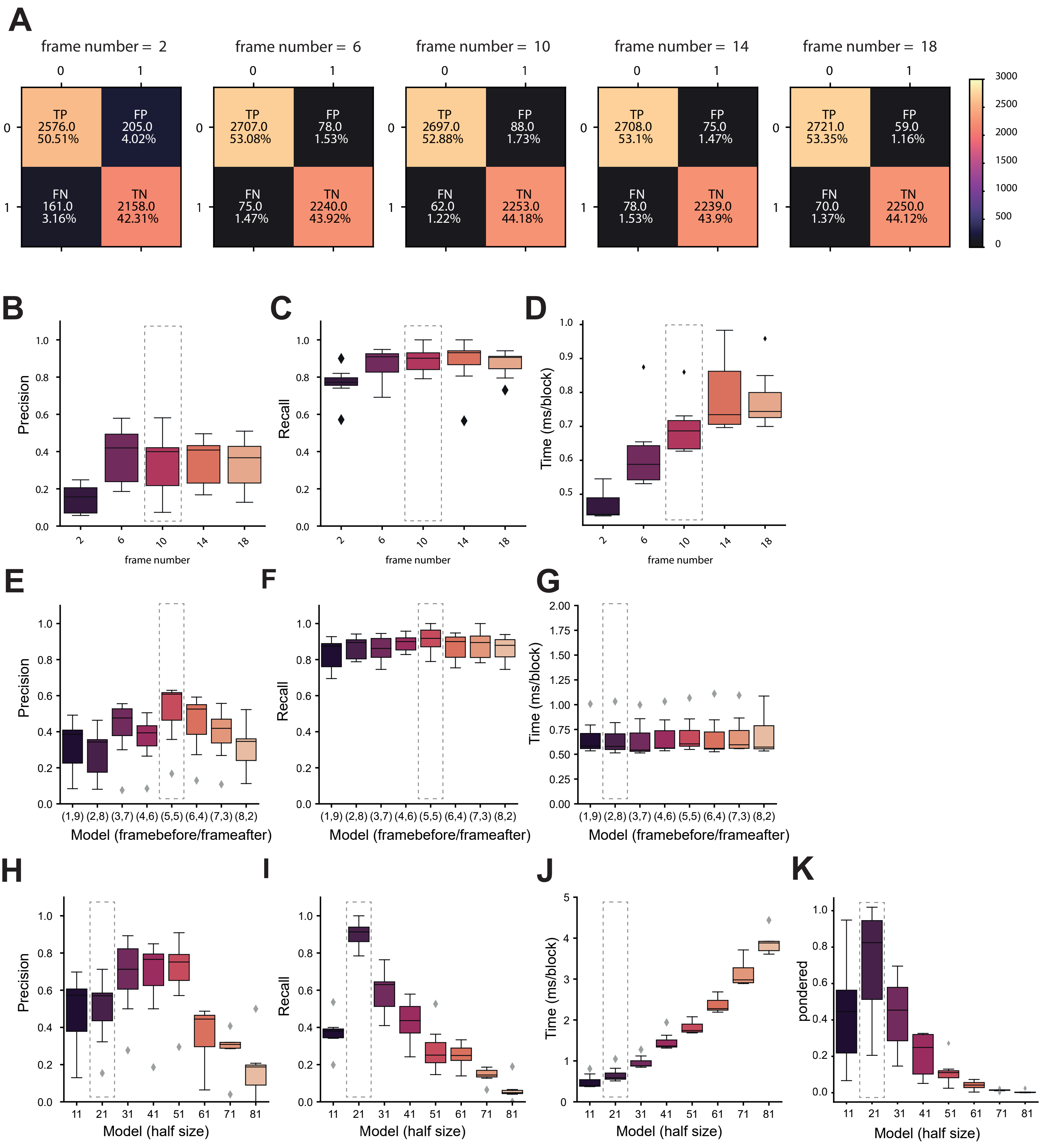
A
frame number = 2
frame number = 6
frame number = 10
frame number = 14
frame number = 18
TP 2576.0 50.51%
FP 205.0 4.02%
FN 161.0 3.16%
TN 2158.0 42.31%
TP 2707.0 53.08%
FP 78.0 1.53%
FN 75.0 1.47%
TN 2240.0 43.92%
TP 2697.0 52.88%
FP 88.0 1.73%
FN 62.0 1.22%
TN 2253.0 44.18%
TP 2708.0 53.1%
FP 75.0 1.47%
FN 78.0 1.53%
TN 2239.0 43.9%
TP 2721.0 53.35%
FP 59.0 1.16%
FN 70.0 1.37%
TN 2250.0 44.12%
B
C
D
Precision
Recall
Time (ms/block)
frame number
E
F
G
Model (framebefore/frameafter)
H
I
J
K
pondered
Model (half size)

Fig. S1. Optimisation of the search window size for extrusion detection

A: Confusion matrix showing the accuracy obtained for different temporal size of the search window (from 2 to 18 time frames). Orange-coloured boxes show the number of correctly predicted events (light orange are True Negatives, darker orange shows True Positives). Grey events are the number of Wrongly predicted events (light grey are False Negative and darker grey show False Positives). The total number of prediction as well as the proportion is given in each case. These values were obtained for a single round of network training for each condition.

B-D: Optimisation of the temporal size of the search window (from 2 to 18 time frames). For each duration, the Precision (**B**), Recall (**C**), and time of calculation (ms per block) (**D**), were estimated on the test dataset. Note that the trainings were not performed on the same set up as in **Figure S1 G**,**J** so the absolute time cannot be compared between these panels. Box plots show the median, the first and third quartile. Top and bottom bars are the maximal and minimal value. Diamonds are outliers.

E-G: Optimisation of the temporal positioning of the search window according to the termination of extrusion (manually detected, end of apical area closure), number of frames before or after the extrusion detection point. For each position, the Precision (**E**), Recall (**F**), and time of calculation (ms per block) (**G**), were estimated on the test dataset. The optimum was obtained for a search window centered on extrusion termination (5,5). Box plots show the median, the first and third quartile. Top and bottom bars are the maximal and minimal value. Diamonds are outliers.

H-K: Optimisation of the size (x y) of the square search window (half size in pixel after rescaling, 1 pixel=0.275 μm). For each position, the Precision (**H**), Recall (**I**), and time of calculation (ms per block) (**J**), were estimated on the training dataset. We computed then a ponderated parameter (**K**, see **Material and Methods**) which takes into account precision, recall and calculation time which peaks for 21px. Box plots show the median, the first and third quartile. Top and bottom bars are the maximal and minimal value. Diamonds are outliers.

Development • Supplementary information

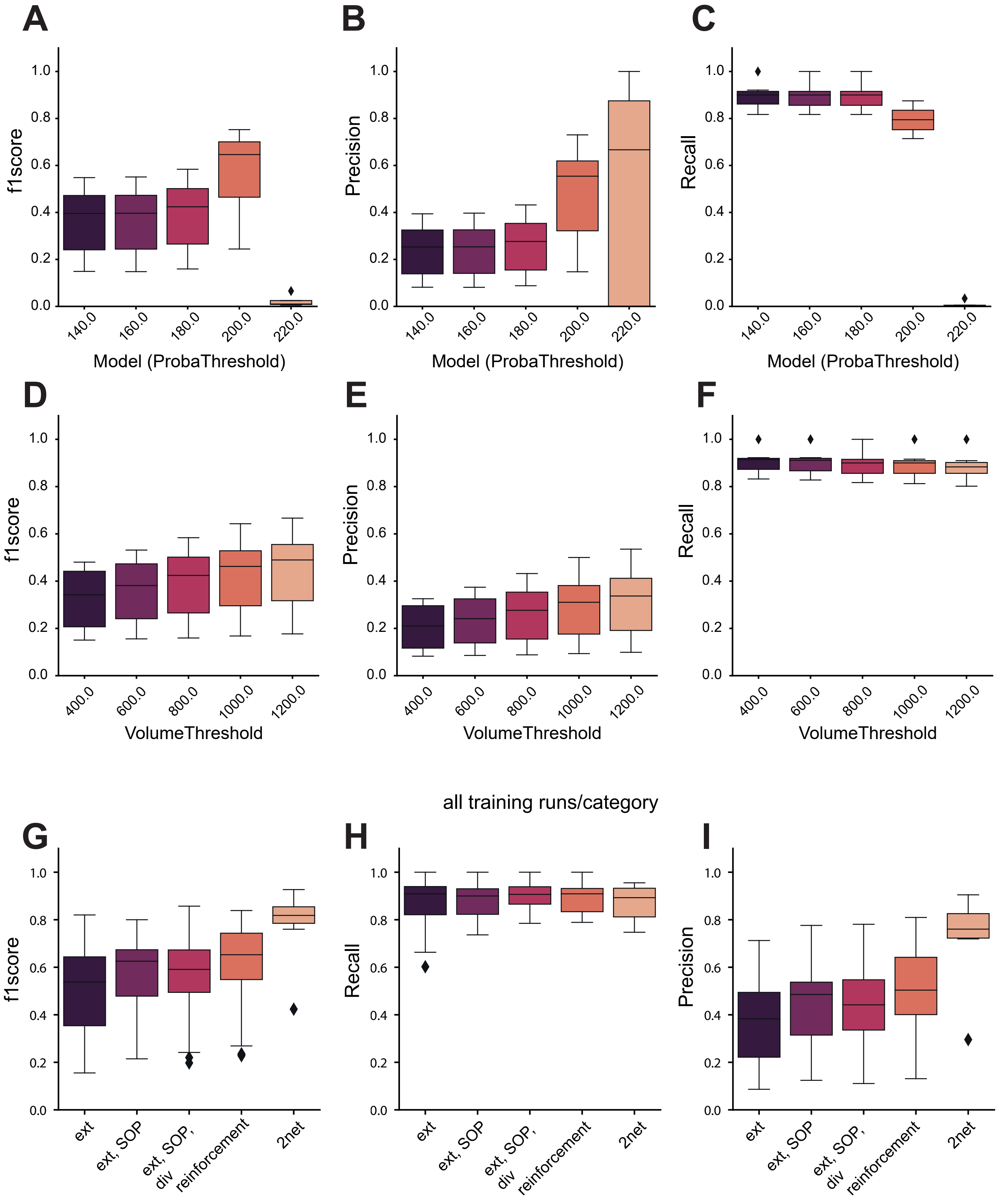

Development • Supplementary information

Fig. S2. Optimisation of probability thresholding for extrusion detection and detection of new categories of cellular events

A-C: Optimisation of the probability threshold used to assign an extrusion event. For each threshold, the f1 score (**A**), precision (**B**), and recall (**C**), were estimated on the test dataset. The optimum f1 score was obtained for 200 (a.u.), however we used 180 in all the rest of our pipeline as we wanted to maximise the recall. Box plots show the median, the first and third quartile. Top and bottom bars are the maximal and minimal value. Diamonds are outliers.

D-F: Optimisation of the threshold probability volume (x-y-t) used to detect extrusion event (in voxel, xy pixel=0.275μm, t=5 minutes). For each volume, the f1 score (**D**), precision (**E**), and recall (**F**), were estimated on the test dataset. The optimium for the f1score was obtained for 1200, however we used a threshold of 800 for all the rest of the pipeline to maximise the recall. Box plots show the median, the first and third quartile. Top and bottom bars are the maximal and minimal value. Diamonds are outliers.

G-I: Changes in the model to optimise its prediction scores on extrusion. **G:** f1-score. **H:** Recall, **I:** Precision, with the initial two class model (ext), the inclusion of SOPs (ext, SOP), the inclusion of SOPs and cell divisions (ext, SOP, div), including the 3 cellular events and reinforcement (see main text), and using two independent networks (2net). The results shown are the compiling of the prediction of all the testing dataset processed through 4 independent trained networks (except for 2net, which used only one pair of networks). Box plots show the median, the first and third quartile. Top and bottom bars are the maximal and minimal value. Diamonds are outliers.

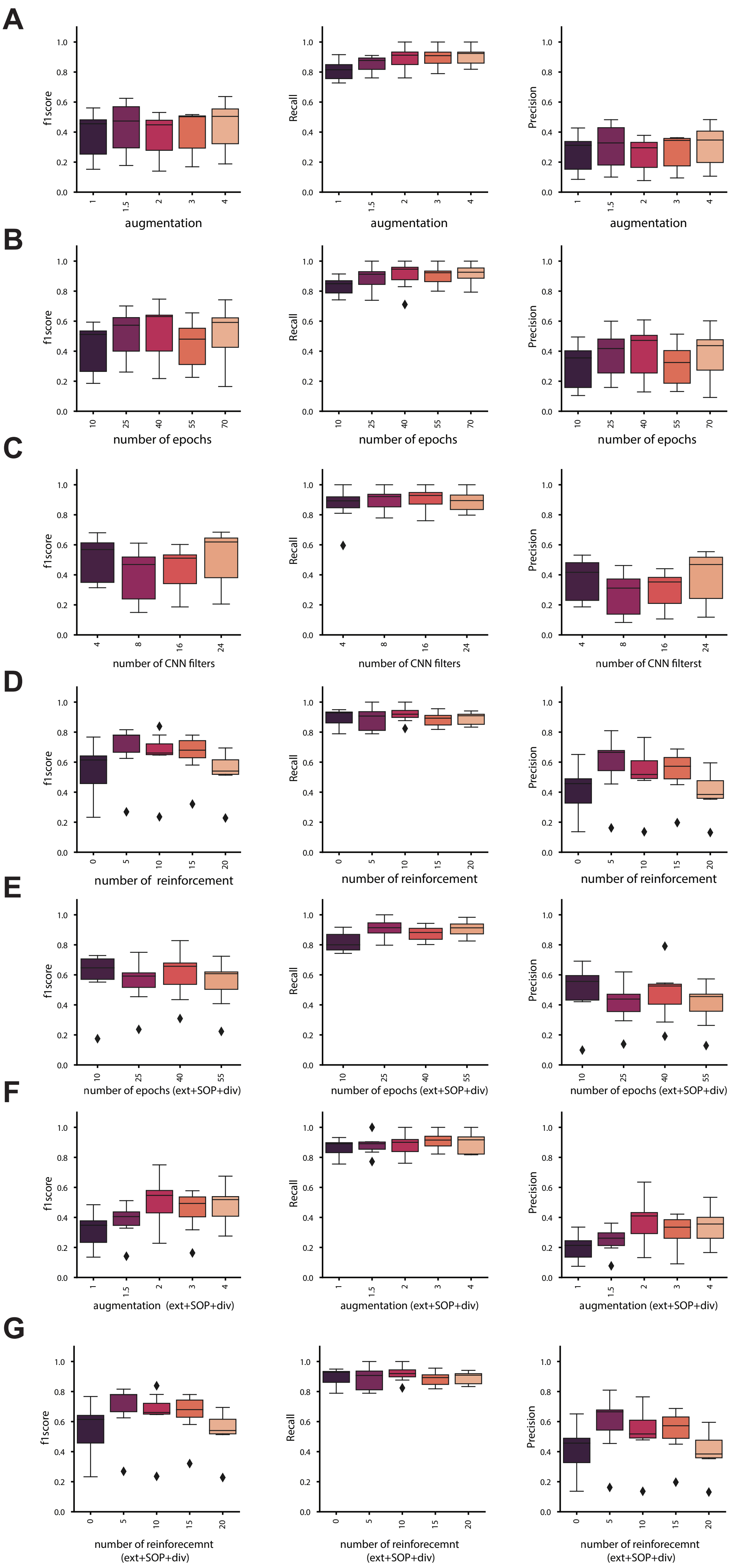
A
B
C
D
E
F
G
f1score
Recall
Precision
1.0
0.8
0.6
0.4
0.2
0.0
augmentation
1
1.5
2
3
4
number of epochs
10
25
40
55
70
number of CNN filters
number of CNN filterst
4
8
16
24
number of reinforcement
number of reinforcement
0
5
10
15
20
number of epochs (ext+SOP+div)
augmentation (ext+SOP+div)
augmentation (ext+SOP+div)
number of reinforecemnt
(ext+SOP+div)

Fig. S3. Optimisation of the hyperparameters of DeXNet

A-F: Optimisation of the hyperparameters of DeXtrusion to maximise the precision and the recall. For each set of parameters, the f1 score, Recall and Precision were estimated on the test dataset either for the DeXNet trained only for extrusion detection (**A-D**) or the DeXNet detecting the four categories of events (extrusion, SOPs, division, control) (**E-G**). The parameters used in this study are summarized in the **Supplementary table 3**. These parameters include the number of augmentations of the data (**A, F**), the number of epochs for model fitting (**B, E**), the number of CNN filters (**C**), and the number of reinforcements (**D, G**). Box plots show the median, the first and third quartile. Top and bottom bars are the maximal and minimal value. Diamonds are outliers.

Table S1. Detailed description of the training dataset

Table summarizing the different properties of the microscopy movies used to train our model. These include the number of the movie in our dataset (Movie column), the type of microscope used (Zeiss LSM880 or Gataca Systems Spinning-Disk), the type of E-cadherin marker used to label the cell boundaries (Marker column), the pixel size and the frame rate. Other genetic modifications or transgenes (other markers, RNAis or drug injections) are described in the 'Genotype' column. Finally, this table also displays the number of events labelled for each movie: extrusions, cell divisions, Sensory Organ Precursors cells (SOPs), Number of false positive detected elements further re-labelled as controls in the pipeline optimisation (Nothing) and total number of elements per movie.

Movie	Microscope	Marker	Pixel size (µm)	Frame rate (seconds)	Genotype	extrusion	Cell division	SOP	Nothing	Total # events/movie
1	LSM 880	ubi-Ecad::GFP	0.1037427	60	UAS-hid-RNAi	36	74	60		170
3	spinning disk	ubi-Ecad::GFP	0.275	300	WT	720	237	279		1236
4	spinning disk	ubi-Ecad::GFP	0.275	300	Gal80ts	223	170	196	47	636
5	spinning disk	ubi-Ecad::GFP	0.275	300	Gal80ts	385	62	266	54	767
6	spinning disk	ubi-Ecad::GFP	0.275	300	Gal80ts SPASTIN	563	96	327	26	1012
8	LSM 880	ubi-Ecad::GFP	0.1037427	60	UAS-hid-RNAi	40	3	37	11	91
9	LSM 880	Ecad::GFP(KI)	0.1037427	60	sqh-mCherry	35	40	33		108
10	LSM 880	Ecad::GFP(KI)	0.1037427	60	sqh-mCherry	40	79	68		187
11	LSM 880	Ecad::GFP(KI)	0.1037427	60	sqh-mCherry	18	100	8	26	152
12	LSM 880	Ecad::tdTomato(KI)	0.1037427	20	jupiter-GFP	20	46	40	6	112
13	LSM 880	Ecad::tdTomato(KI)	0.1037427	20	jupiter-GFP	34	117	99	6	256
14	LSM 880	Ecad::tdTomato(KI)	0.1037427	20	SAS4-GFP	59	36	103	9	207
16	LSM 880	Ecad::tdTomato(KI)	0.1037427	300	colcemid	215		45		260
17	LSM 880	Ecad::tdTomato(KI)	0.1037427	300	colcemid	203				203
19	spinning disk	Ecad::tdTomato(KI)	0.275	300	UAS-hid-RNAi ctrl inj	104	123	60	22	309
20	spinning disk	Ecad::tdTomato(KI)	0.275	300	UAS-hid-RNAi ctrl inj	82	116	80	5	283
21	spinning disk	Ecad::tdTomato(KI)	0.275	300	UAS-hid-RNAi ctrl inj	77	487	109	10	683
23	spinning disk	Ecad::GFP(KI)	0.275	300	Gal80ts SPASTIN	566	40	297		903
24	spinning disk	Ecad::GFP(KI)	0.275	300	Gal80ts SPASTIN	464	305	166	25	960
25	LSM 880	Ecad::GFP(KI)	0.18	300	WT	486	331	103		920
27	LSM 880	Ecad::GFP(KI)	0.18	300	WT	878	260	165		1303
28	LSM 880	Ecad::GFP(KI)	0.18	300	EGFR-RNAi	435	60	107	26	628
29	LSM 880	Ecad::GFP(KI)	0.18	300	EGFR-RNAi	489	77	174		740
31	spinning disk	Ecad::GFP(KI)	0.275	300	EGFR-RNAi	528	162	232	31	953
					Total # events/category	6700	3021	3054	304	

Development • Supplementary information

Table S2. Detailed description of the test dataset

Table summarizing the different properties of the microscopy movies used to test our model. These include the number of the movie in our dataset (Movie column), the type of microscope used (Zeiss LSM880 or Gataca Systems Spinning-Disk), the type of E-cadherin marker used to label the cell boundaries (Marker column) , the pixel size and the frame rate. Other genetic modifications or transgenes (other markers, RNAis or drug injections) are described in the 'Genotype' column. Finally, this table also displays the number of events labelled for each movie: extrusions, cell divisions, Sensory Organ Precursors cells (SOPs) and total number of elements per movie.

Movie	Microscope	Marker	Pixel size (μm)	Frame rate (seconds)	Genotype	Extrusions	Divisions	SOPs	Total # events/movie
2	spinning disk	Ecad::GFP(KI)	0.275	300	WT	883	1351	351	2585
7	spinning disk	ubi-Ecad::GFP	0.275	300	UAS-hid-RNAi	11	26	132	169
15	LSM 880	Ecad::tdTomato(KI)	0.1037427	20	WT	63	89	146	298
18	LSM 880	Ecad::tdTomato(KI)	0.1037427	300	UAS-hid-RNAi colcemid	123			123
22	spinning disk	Ecad::tdTomato(KI)	0.275	300	UAS-hid-RNAi ctrl inj	59	46	193	298
26	LSM 880	Ecad::GFP(KI)	0.18	300	WT	682	120	130	932
30	LSM 880	Ecad::GFP(KI)	0.18	300	EGFR-RNAi	499	108	100	707
					Total events #/category	2320	1740	1052	5112

Table S3. Optimisation of model hyperparameters

Table describing the final hyperparameter values used in the final pipeline after careful optimisation and their associated F1-score values. We first optimised the number of augmentation, epochs and CNN filters in the training including extrusions only (ext. only rows). Then we fixed the numbers of CNN filters for the rest of the training. We then included cell divisions and SOPs in order to optimise the pipeline and explored the impact of the number of augmentation and epochs on the F1-score (4 classes rows). Finally, we further optimised the model by adding false positive detected elements further re-labelled as controls in the pipeline optimisation and check the impact on the F1-score (this was done only in the case of 4classes training).

	Hyperparameter	Parameter value	Optimal F1-score
ext. only	Number of augmentation	4	0.504653
	Number of epochs	40	0.630638
	Number of CNN filters	24	0.618855
4 classes	Number of augmentation	2	0.546644
	Number of epochs	40	0.657036
	Number of reinforcement	5	0.779413

Table S4. Generalisation scores on unseen movies

Table summarising the generalisation scores (precision and recall) depending on the movie the generalisation was tested on. For each movie checked the scores with the model without further training (out-of-the-box rows) or after retraining (retrained rows). For Larval Epithelial Cells movies (LECs rows) we proceeded to an additional retraining step by including movies from this dataset (retraining with 2 movies).

		Generalisation scores	
		Precision	Recall
UAS-EGFR-RNAi	Out-of-the-box	0.874	NA
	Retrained	NA	NA
Pupal wing	Out-of-the-box	0.786	0.849
	Retrained	0.909	0.83
LECs	Out-of-the-box	0.738	0.38
	Retrained	0.858	0.69
	Retrained with 2 movies	0.860	0.74

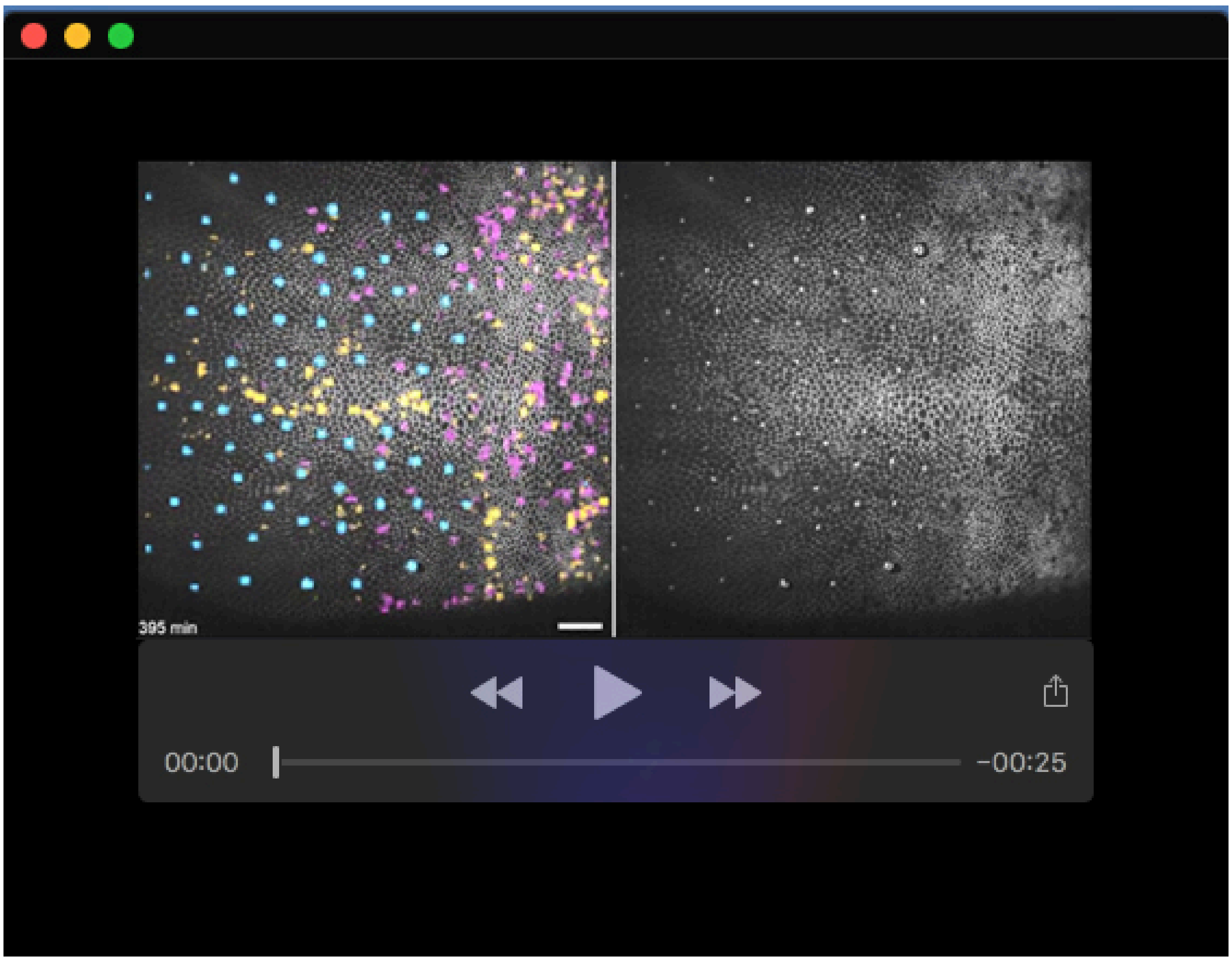

Movie 1. Probability map of extrusions, SOPs and divisions in a WT pupal notum

Local projection of a pupal notum expressing E-cad::GFP (grey) overlayed with the probability map of detection of extrusions (yellow), cell divisions (magenta) and SOPs (cyan). E-cad channel is shown separated on the right. Anterior, left and posterior right. Scale bar=30μm.

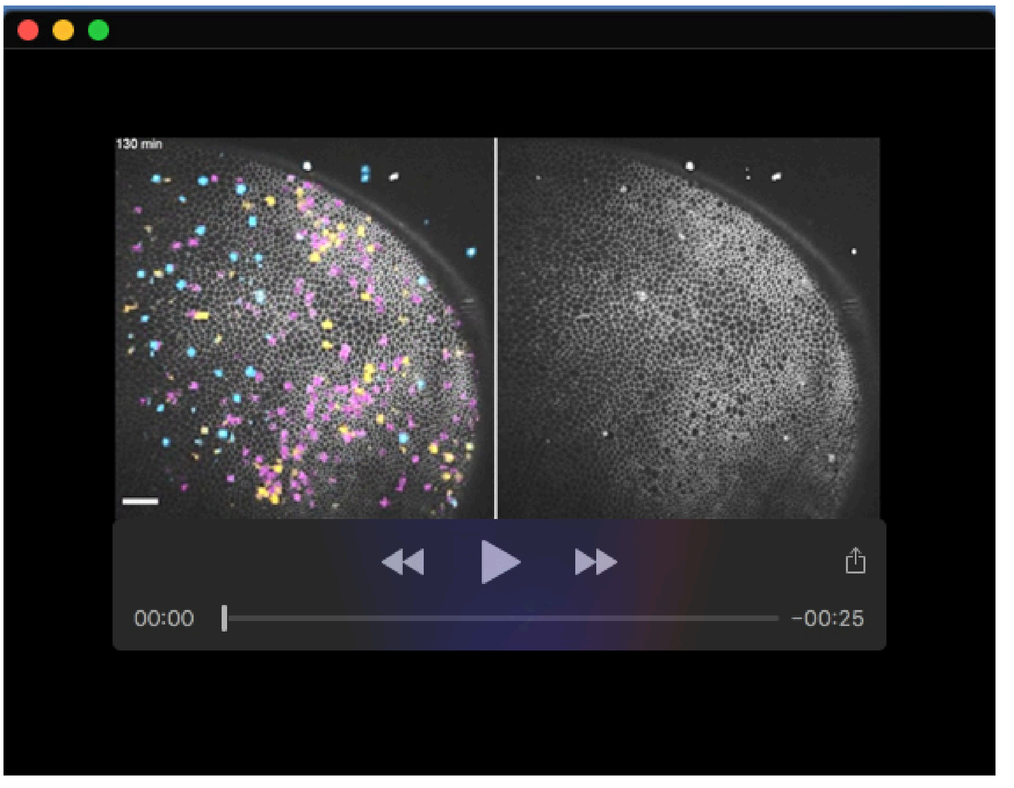

Movie 2. Probability map of extrusions, SOPs and divisions in an EGFR depleted pupal notum

Local projection of a pupal notum depleted for EGFR (*pnr-Gal4, UAS-EGFRdsRNA*) expressing E-cad::GFP (grey) overlayed with the probability map of detection of extrusions (yellow), cell divisions (magenta) and SOPs (cyan). E-cad channel is shown separated on the right. Anterior, left and posterior right. Scale bar=30μm.

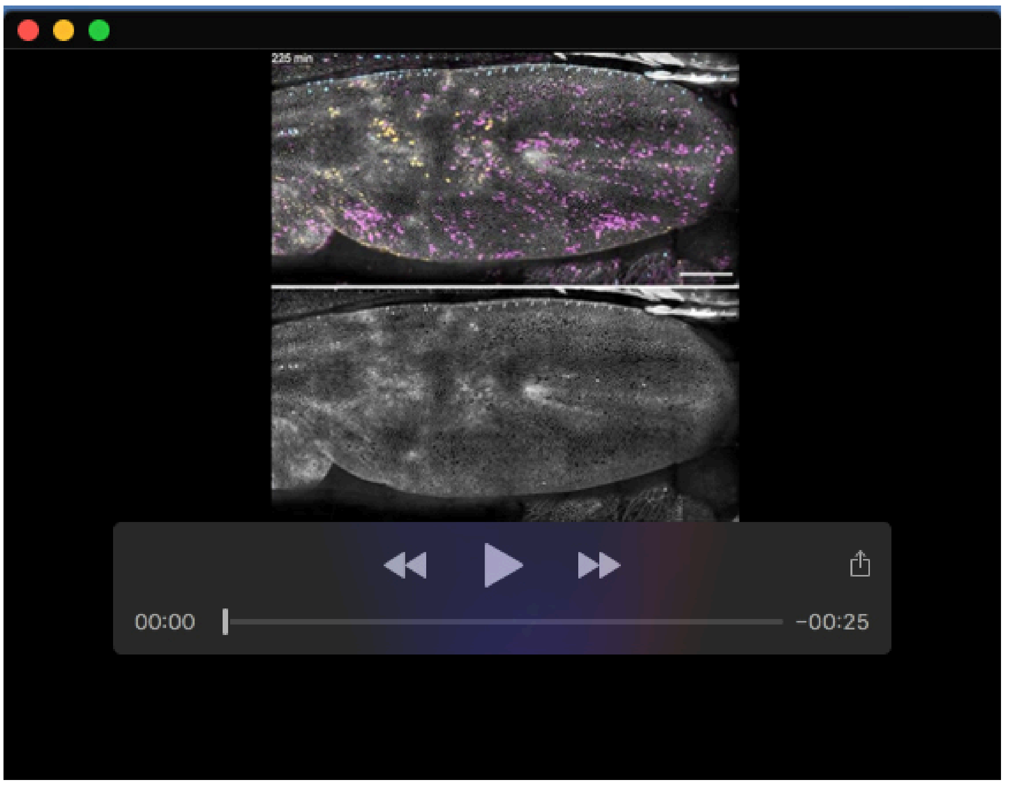

Movie 3. Probability map of extrusions, SOPs and divisions in a pupal wing

z-projection of a WT pupal wing expressing E-cad::GFP (grey) from (Etournay et al., 2015), overlayed with the probability map of detection of extrusions (yellow) and cell divisions (magenta). E-cad channel is shown separated on the bottom. Distal on the right, proximal on the left. Scale bar=50μm. Note that we only took in consideration the probability overlapping the wing.

Development • Supplementary information

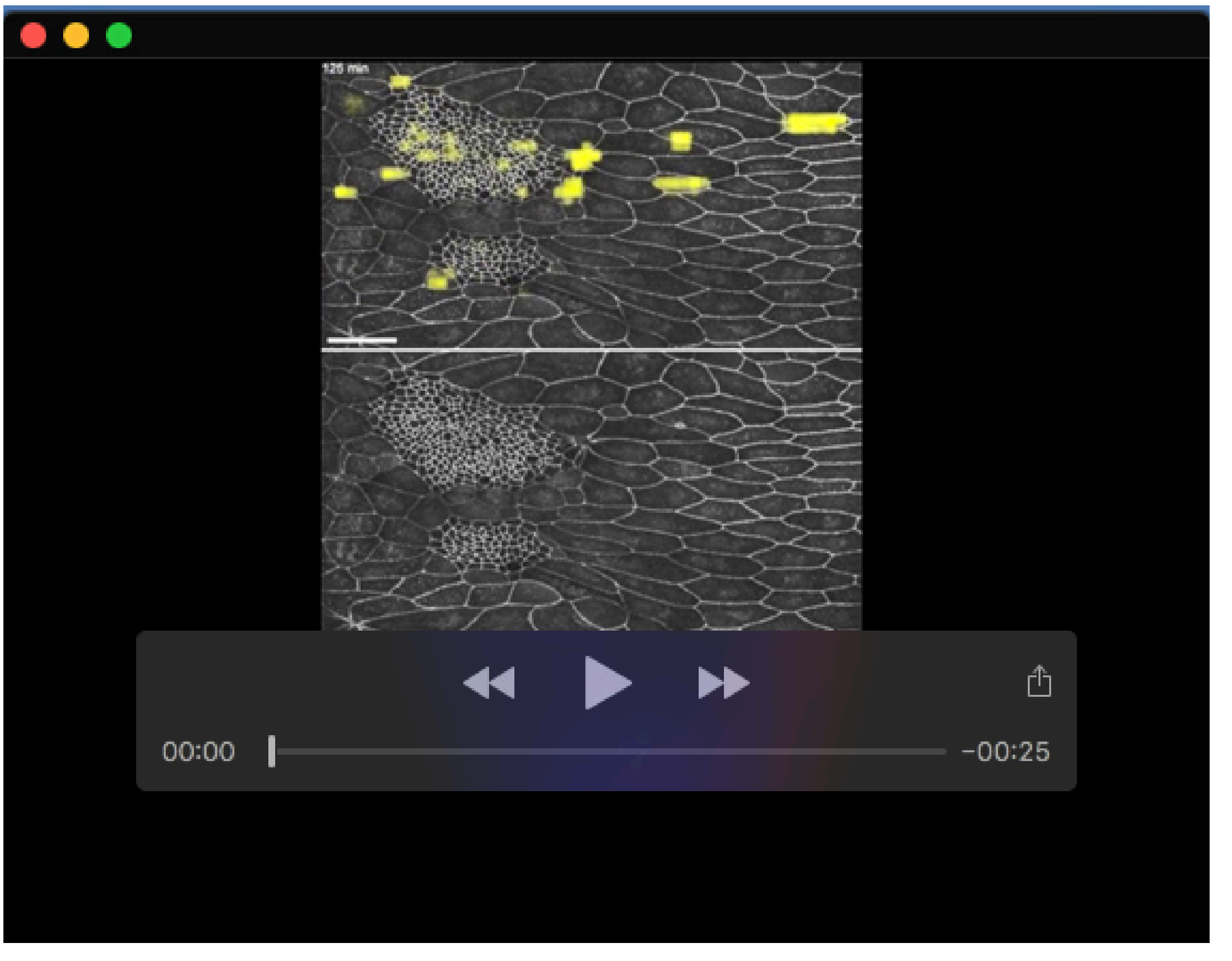

Movie 4. Probability map of extrusions in the larval epidermal cells of the pupal abdomen

z-projection of a WT pupal abdomen expressing E-cad::GFP (grey) from (Davis et al., 2022) overlayed with the probability map of detection of extrusions (yellow). E-cad channel is shown separated on the bottom. Scale bar=50μm. Note that we only used the prediction in the LECs and ignored the histoblasts (small cells in the clusters on the left). The cell scale used for this prediction is suboptimal for histoblasts.
