# Supplementary material for: DeXtrusion: automatic recognition of epithelial cell extrusion through machine learning in vivo
Source: Development. 2023 Jun 30;150(13):dev201747. doi: 10.1242/dev.201747 (PMC10323232; doi:10.1242/dev.201747)
Supplement: Supplementary file 1 [file develop-150-201747_review_history.pdf]

DeXtrusion: automatic recognition of epithelial cell extrusion through machine learning *in vivo*

Alexis Villars, Gaëlle Letort, Léo Valon and Romain Levayer
DOI: 10.1242/dev.201747

Editor: Paul Francois

Review timeline
Original submission: 3 March 2023
Editorial decision: 25 April 2023
First revision received: 17 May 2023
Accepted: 28 May 2023

Original submission

First decision letter

MS ID#: DEVELOP/2023/201747

MS TITLE: DeXtrusion: Automatic recognition of epithelial cell extrusion through machine learning in vivo

AUTHORS: Alexis Villars, Gaële Letort, Léo Valon, and Romain Levayer

I have now received all the referees reports on the above manuscript, and have reached a decision. The referees' comments are appended below, or you can access them online: please go to BenchPress and click on the 'Manuscripts with Decisions' queue in the Author Area.

The overall evaluation is positive and we would like to publish a revised manuscript in Development, provided that the referees' comments can be satisfactorily addressed. Please attend to all of the reviewers' comments in your revised manuscript and detail them in your point-by-point response. If you do not agree with any of their criticisms or suggestions explain clearly why this is so. If it would be helpful, you are welcome to contact us to discuss your revision in greater detail. Please send us a point-by-point response indicating your plans for addressing the referee's comments, and we will look over this and provide further guidance.

Reviewer 1

Advance summary and potential significance to field

See pdf

Comments for the author

Review report of the manuscript "DeXtrusion: Automatic recognition of epithelial 1 cell extrusion through machine learning in vivo"

Villars and Letort et al. present DeXtrusion, a machine learning-based method for automated detection of epithelial cell extrusion events as an example of spatiotemporally regulated cellular processes. With the emergence of large-scale biology, with automation that enables high-content

© 2023. Published by The Company of Biologists under the terms of the Creative Commons Attribution License (https://creativecommons.org/licenses/by/4.0/).

data collection, the limiting factor for systematic characterization of spatiotemporal cellular events, is the ability to effectively and accurately measure these events in time and space, which currently relies on tedious manual detection/correction. DeXtrusion is formulated as a binary classification problem. A recurrent convolutional neural network receives as input an image region ("window") through the time sequence and outputs a prediction (classification) to whether an extrusion event occurred. DeXtrusion can be applied directly using the raw image data and does not require precise segmentation and tracking. The authors train their network using manually annotated in vivo live imaging of the Drosophila pupal notum marked with fluorescent E-cadherin. To deal with the high volume of false-positive detection, the authors refine their network to filter SPO and division events that were erroneously classified as extrusions. This revised model performed well, and was shown to be generalized, or "transferred", to other live imaged epithelial tissues with cell junctional markers without requiring extensive retraining. The authors' report that DeXtrusion leads to a ~10 fold improvement (from ~10 hours to ~1 hour) in the time required for manual annotations versus corrections respectively. A similar approach can be taken to other spatiotemporal cellular events such as cell differentiation/proliferation/death.

The strengths of the manuscript are being one of the first focusing on automated identification of spatiotemporal cellular events that are important for precise regulation of developmental processes. I especially liked the practical aspect of reporting the time potentially saved by using an automated approach followed by manual correction/filtering, which is not common in such technical papers. I also liked the authors' efforts on assessing DeXtrusion on unseen tissues. While I make a specific proposal regarding presentation (see below), the manuscript was relatively easy to follow (except a few technical details mentioned below). The manuscript is accompanied with open code (looks well organized and documented - I did not try to execute it). In general, I do not have any major objection against publication of this article, which is likely to make a nice contribution to the field.

Major comments and suggestions:

1. A small fraction of false-positives can lead to major detection mistakes: identifying many false events that can dramatically skew the statistics. This was a major concern for me while reading the first parts of the manuscript (staring in line #176 reporting 2% of false positive detection), a concern that was resolved only in Fig. 3. This was very confusing to me, for example, in Fig. 2E-G one can observe the high volume of false-positive (which are not referred to explicitly), however these false-positive are not reported in Fig. 2G-H.

I suggest the following writing adjustments to make this point clear: (1) Explicitly explain and demonstrate the problem of false positives in imbalanced detection problems as early as possible (even in the context of Fig. 1). Remember that Development's readers are not necessary computational savvy, and this disclaimer is even more important for potential users. (2) The first parts of the manuscript could be shorten brining the results of Fig. 3 earlier (IMO, not all panels of Fig. 2 are necessary - maybe even consider merging Figures 1-2 to a single figure?).

2. How important is time? Why was a time window of 10 frames selected? How critical is time for the performance and how sensitive is this parameter? Since the method is focused on dynamic events, I think that a comprehensive assessment (by changing the time window considered) is in place. I am aware that Fig. S1 shows different positioning of the time window, but it does not provide information regarding how important is time, and what are the effective time ranges for prediction of extrusion events.

3. The model adjustments to filter false-positive were not sufficiently clear to me. This is described in lines #240-242 and in Fig. 3F and its legend. My understanding is that a new model was trained with false-positives classified to SOP, division, or control events, in addition to the previous events, followed by another round of training with inclusion of false-positives (lines #250-252). If this is the case, please better explain that in the main text, figure legend, and Methods. Another question is how generalizable/practical/efficient is this type of re-training based on manually annotated false-positive observations? I think that this point should be further clarified in the Discussion.

© 2023. Published by The Company of Biologists under the terms of the Creative Commons Attribution License (https://creativecommons.org/licenses/by/4.0/).

Other comments and suggestions:

1. Line #149: "since CNN on single images performed poorly at predicting extrusion" - can you show this result for comparison?
2. Fig. 2G-H are not referenced from the main text.
3. Instead of reporting size in pixels (e.g., line #184, Fig. 2D-E, line #265) or time in frames (e.g., Fig. 2D,F, Fig. S1C, line #524), please report in microns and minutes correspondingly throughout the manuscript.
4. I could not find the Table legends.
5. Line #300: using manually annotated extrusion and division events from the dataset used for analysis is not problematic in terms of "training data contamination"?
6. I wonder how would the performance of a model that uses cell shape dynamics compared to DeXtrusion that uses the raw image data. This can be achieved without long-term tracking, rather using short trajectories (that are not so prune to mistakes) and training a model from shape features extracted from these trajectories. This would extend the scope of the study, and not necessary to support the main claims/conclusion.

Minor cosmetic suggested changes/typos

1. Line #41, extra space at the end of the sentence.
2. Line #58, missing "a" ("in a tissue").
3. Fig. 1A - mark the number of events in the histogram
4. In Tables S1 and S2 change the label "death" to "extrusion"
5. Fig. 2 legend: H ' G, I ' H
6. Fig. 4 legend - the colors do not fit to the figure (green ' gray, magenta ' green, light purple ' light green)
7. Line #503: "…)".

Assaf Zaritsky,
Ben-Gurion University of the Negev, Israel

Reviewer 2

Advance summary and potential significance to field

In this manuscript, Villars, Letort, and their team have developed a tool named DeXtrusion that utilizes neural networks to automatically detect biological events, such as cell extrusion, cell division, and cell differentiation (SOP formation) in a confocal fluorescent video of a Drosophila epithelium. Impressively, DeXtrusion has been successful in reducing the time required for annotation by 10-fold with high precision. Moreover, DeXtrusion has demonstrated its applicability to other epithelial tissue, such as pupal wing and pupal abdomen, making it a valuable and versatile tool for the scientific community. Overall, I recommend the publication of this manuscript in Development, provided that several points, which are primarily clarifications and presentations, are addressed. I listed these points below.

Comments for the author

MAJOR:

#1) After reading the manuscript, I was initially confused about the different functions DeXtrusion and DeXNet. To avoid this confusion, I suggest that the authors make the following changes:
- In Fig. 1C, replace the wording “DeXtrusion model” with “DeXNet (model)”, if that is what is meant.
- Before diving into the details of the models (line 141), explain the relationship between DeXtrusion and DeXNet. My understanding is that DeXNet is the model used to detect the events in a cropped video, while DeXtrusion is the overall pipeline used to apply DeXNet to the entire time and space of the video.

© 2023. Published by The Company of Biologists under the terms of the Creative Commons Attribution License (https://creativecommons.org/licenses/by/4.0/).

Clarifying this relationship at the outset will aid in better understanding the subsequent model descriptions.

#2) Fig. 1C-C'.
The manuscript describes how temporal information history was incorporated using a recurrent neural network architecture. However, it is not clear in Fig.1C which part of the image indicates the temporal history.
- Similar presentation in Fig. 2A can be applied here, too.
- It is also unclear whether the "10@" in "10@45x45" refers to the time sequence. To avoid confusion, the authors should elaborate on what this notation "10@" means. The same goes for "1@", "8@", and "64@" in Figure 1C.
- Is "10@64" before GRU in Fig. 1C supposed to be "10@1x64"?
- You may consider making the 1x64 parallelogram in Fig. 1C' into a rectangle like the yellow rectangle used before GRU (10@64).
- It would be helpful to add a few sentences describing CNN (Fig. 1C') in the text.

#3) I have a different impression between Fig. 2D and Fig. 2E&F, particularly with regards to the temporal information. This could be due to the difference in the binning used in the figure. Therefore, I recommend that the author apply the same binning as used in Fig. 2D to Fig. 2F.

MINOR:

#4) It would be helpful to describe how each event was manually annotated. For instance. Specifically, which software was used, and which time point (or duration?) was used to define each event, such as extrusion?

#5) It would be helpful to give a number or range of "the temporal size of the sequences (line 166)".

#6) Some of the terminologies were not well-defined, particularly for potential readers of the journal who may not be familiar with the specific terminology. I recommend that the authors provide more precise and clear definitions for the following words in the text:
- "loss" in Fig. 1D.
- "recall" in Fig. 3E', S1, S2, and S3.
- "recall" and "precision". What does "real events" (line 218) mean?

#7) What's the pixel resolution of the "final local probability output (line 191)"? Is it 22x22 pixels? It would be helpful to describe this in the text.

#8) It would be helpful to describe how the manual annotation was done particularly whether the annotation included the time duration. If not, it would be useful to clarify which time point was considered as an "extrusion", "division", and "SOP".

#9) To be accurate, "4 time frames (line 215)" could be "+/- 4 time frames". Just for your consideration.

#10) Microscope images in Fig. 3F are too small.

#11) The main text didn't explain the difference between "ext, SOP" vs "extr, SOP div" shown in Fig. 3E-E". Please expand the text.

#12) "extr" in Fig. 3E-E" could be "ext".

© 2023. Published by The Company of Biologists under the terms of the Creative Commons Attribution License (https://creativecommons.org/licenses/by/4.0/).

First revision

Author response to reviewers' comments

“DeXtrusion: Automatic recognition of epithelial cell extrusion through machine learning *in vivo*”

General comments

We would like to thank the reviewers for their very constructive comments which helped to improve the readability and accuracy of our article. We have addressed all the points raised by the referees while trying to find a good compromise suitable for expert and non-expert readers. We decided to maintain the main organisation of figures as it helps to decompose the pipeline, while clearly indicating in the main text the problem of false positives early on. We have also included now some quantifications on the impact of the size of the temporal window for events detection showing that prediction accuracy drastically drops down when reducing the number of time frames. We have also extended the discussion by introducing future developments (inclusion of geometrical features through segmentation and generalisation) as well as the generalisation of the reinforcement procedure.

All the modifications in the main text are highlighted in blue.

Point by point answer to reviewers

Reviewer 1:
Review report of the manuscript “DeXtrusion: Automatic recognition of epithelial 1 cell extrusion through machine learning in vivo”

Villars and Letort et al. present DeXtrusion, a machine learning-based method for automated detection of epithelial cell extrusion events as an example of spatiotemporally regulated cellular processes. With the emergence of large-scale biology, with automation that enables high-content data collection, the limiting factor for systematic characterization of spatiotemporal cellular events, is the ability to effectively and accurately measure these events in time and space, which currently relies on tedious manual detection/correction. DeXtrusion is formulated as a binary classification problem. A recurrent convolutional neural network receives as input an image region (“window”) through the time sequence and outputs a prediction (classification) to whether an extrusion event occurred. DeXtrusion can be applied directly using the raw image data and does not require precise segmentation and tracking. The authors train their network using manually annotated in vivo live imaging of the Drosophila pupal notum marked with fluorescent E-cadherin. To deal with the high volume of false-positive detection, the authors refine their network to filter SPO and division events that were erroneously classified as extrusions. This revised model performed well, and was shown to be generalized, or “transferred”, to other live imaged epithelial tissues with cell junctional markers without requiring extensive retraining. The authors’ report that DeXtrusion leads to a ~10 fold improvement (from ~10 hours to ~1 hour) in the time required for manual annotations versus corrections respectively. A similar approach can be taken to other spatiotemporal cellular events such as cell differentiation/proliferation/death.

The strengths of the manuscript are being one of the first focusing on automated identification of spatiotemporal cellular events that are important for precise regulation of developmental processes. I especially liked the practical aspect of reporting the time potentially saved by using an automated approach followed by manual correction/filtering, which is not common in such technical papers. I also liked the authors’ efforts on assessing DeXtrusion on unseen tissues. While I make a specific proposal regarding presentation (see below), the manuscript was relatively easy to follow (except a few technical details mentioned below). The manuscript is accompanied with open code (looks well organized and documented – I did not try to execute it). In general, I do not have any major objection against publication of this article, which is likely to make a nice contribution to the field.

Answer: We thank the reviewer for the very positive comments on the usefulness of our approach

© 2023. Published by The Company of Biologists under the terms of the Creative Commons Attribution License (https://creativecommons.org/licenses/by/4.0/).

and our effort to make it accessible.

Major comments and suggestions:
1. A small fraction of false-positives can lead to major detection mistakes: identifying many false events that can dramatically skew the statistics. This was a major concern for me while reading the first parts of the manuscript (staring in line #176 reporting 2% of false positive detection), a concern that was resolved only in Fig. 3. This was very confusing to me, for example, in Fig. 2E-G one can observe the high volume of false-positive (which are not referred to explicitly), however these false-positive are not reported in Fig. 2G-H. I suggest the following writing adjustments to make this point clear: (1) Explicitly explain and demonstrate the problem of false positives in imbalanced detection problems as early as possible (even in the context of Fig. 1). Remember that Development's readers are not necessary computational savvy, and this disclaimer is even more important for potential users. (2) The first parts of the manuscript could be shorten brining the results of Fig. 3 earlier (IMO, not all panels of Fig. 2 are necessary – maybe even consider merging Figures 1-2 to a single figure?).

Answer: Thank you for this very relevant suggestion. It is true that we openly discuss about the false positive only in Figure 3 as we wanted to decompose before the core of the network. As suggested, we now explain very early the issue. Regarding point (1) and imbalanced detection, we have indeed taken into account this issue by adjusting the number of overrepresented events in the training dataset (mostly by removing sequences with no events). This was explicitly included in the methods in the "Data imbalance" section. Our main motivation was to optimize the recall (extrusion detection), so that the user only has to check the correctness of the detections, which is faster than looking at the movie for an event. For clarity, we now discuss this point early on in the first section of the main text (line 170):

*"To avoid imbalanced detection in our training dataset (due to the over-representation of "no event" sequences relative to extrusion), we have equalised the number of extrusion and control sequences used for the training (see **Material and Methods**)."*

Having said that, the 2% of false positives found in our training dataset still constitute a challenge as pointed out by the referee. Given the larger proportion of "no event" sequences in the full movie, we expected to have a significant number of false positives (a problem that we solved later in the article by optimising the precision). We now clearly point out this problem early on in the first section line 195:

"Of note, these 2% of false positives could constitute a challenge for detection in the full movies since the proportion of "no event" sequences is much higher than the proportion of real extrusion. This may generate a significant proportion of false positives (thus reducing our "precision"). We will discuss the optimisation of the precision in later sections."

Regarding the reduction of the text, we understand the point, but at the same time feel that merging Figures 1 and 2 will generate a very heavy figure. While the separation between Figure 1, 2 and 3 is admittedly a bit artificial (especially when using the full DeXtrusion pipeline for comparing precision, recall and F1 score for different model parameters in Figure S1), we would prefer to keep this decomposition as it really helps to understand the process even for non-specialist readers (clearly focusing first on DeXNet, then DeXtrusion and full movie detection and finally precision optimisation). We hope this will be fine now that we introduced the problem of false positives early on in the text.

2. How important is time? Why was a time window of 10 frames selected? How critical is time for the performance and how sensitive is this parameter? Since the method is focused on dynamic events, I think that a comprehensive assessment (by changing the time window considered) is in place. I am aware that Fig. S1 shows different positioning of the time window, but it does not provide information regarding how important is time, and what are the effective time ranges for prediction of extrusion events.

Answer: Thanks for raising this very important point. Indeed, we have not explored extensively the impact of the number of frames on our prediction in the first version of the manuscript. Our very first tries of cell extrusion recognition were based on single time points and were not

© 2023. Published by The Company of Biologists under the terms of the Creative Commons Attribution License (https://creativecommons.org/licenses/by/4.0/).

successful at all. This was based however on a completely different pipeline and we have not really documented this quantitatively (this was mostly an exploration phase of the project). This is somehow quite intuitive as extrusions are mostly detected not through specific cell shapes or signals, but rather by the dynamics of constriction and cell shape changes. Regarding the time frames, we picked 10 frames (for a total of 50 minutes) as extrusion events typically last 20 to 30 minutes in the pupal notum (with a slight skew distribution, see Villars et al., Nat. Comm. 2022 in Figure 6o), as such, a 50 minutes window will cover the vast majority of extrusion from the beginning (start of apical constriction) to the disappearance of apical area. Taking a longer time window increases the chance to catch two consecutive extrusions in the same cropped sequence and increases computation time. We now clearly discuss this point in the main text line 182:

*"For instance, the temporal size of the sequences (10 frames, 50 minutes) was chosen so that it contains enough information to recognise extrusion (lasting on average 20 to 30 minutes (Villars et al., 2022)) while reducing the chance to capture two events and reducing calculation time (see **Material and Methods**, **Fig. S1A-D**)."*

As suggested, we have also now explored the impact of the number of frames on the prediction from using two time-frames to 18 time-frames (**see new Figure S1A-D**). Of note, due to the architecture of the network, we could not process a single time frame. Interestingly, the accuracy of the confusion matrix drastically drops when reducing the number of frames to two (**Figure S1A**), as well as the precision and recall (**Figure S1 B,C**), thus demonstrating the important role of temporal information. Precision and recall reach a maximum at around 6-10 time-frames and were not drastically changed by increasing the size of the temporal windows. This suggests that our 10 time-frame selection is a good compromise between precision/recall and calculation time. We mention this point in line 186:

*"Importantly, we observed a drastic reduction of the precision and recall when restraining the time windows to two frames, illustrating the key role of temporal information for accurate detection (**Fig. S1 A-D**)."*

3. The model adjustments to filter false-positive were not sufficiently clear to me. This is described in lines #240-242 and in Fig. 3F and its legend. My understanding is that a new model was trained with false-positives classified to SOP, division, or control events, in addition to the previous events, followed by another round of training with inclusion of false-positives (lines #250-252). If this is the case, please better explain that in the main text, figure legend, and Methods. Another question is how generalizable/practical/efficient is this type of re-training based on manually annotated false-positive observations? I think that this point should be further clarified in the Discussion.

Answer: We apologise if this was not clear. Indeed, as accurately described by the referee, we have first included the new classes (SOPs, divisions) and then retrained the network. This was then followed by the identification of false positives which were tagged as "no event" in the training dataset. We have now better described the procedure in the main text line 274:

"To further enhance our precision, we then manually screened all remaining false positive detections and included image sequences representative of the patterns of these false positives in the training inputs as control sequences and retrained the DeXNet network (using the network including the four classes of events)."

We have also reworded the legend of Figure 3 to make it clearer hopefully.

Regarding the generalisation of the reinforcement procedure, it is actually hard to judge for any other type of event detection. In our case, the reinforcement and inclusion of new cellular events procedure were triggered by the observation of the false positive detections. Typical false detections were cellular events (division and SOPs mainly), or transient cell deformations restricted to few time points, or small bright points visible on a few frames. The last two typical false positives were added as "control" events in the retraining as they are not per se well defined cellular events (as far as we can judge). The inclusion of the false positive in the retraining led to a significant improvement of the f1 score (from ~0.5 to 0.7, **Figure 3E**) mostly due to an increased precision. Globally, the combined effect of the classification of new cellular events, reinforcement

© 2023. Published by The Company of Biologists under the terms of the Creative Commons Attribution License (https://creativecommons.org/licenses/by/4.0/).

of the training and combined network increased the precision by a factor of 3. As suggested, we now clearly discuss the relevance of this reinforcement in the discussion and suggest that it could be easily applied to any other pipeline of event detection, line 406:

"Moreover, the high rate of false positives of our initial pipeline revealed the difficulty of detecting one particular type of un-frequent event in a movie containing potentially other events. The strategies we applied to improve the precision (classifying other cellular events as well, reinforcement of the training by including other typical false positives ,e.g. transient cell deformation, and averaging the prediction of several networks) led to a drastic increase in the prediction precision (~3-folds increase). To our knowledge, these methods were not so commonly used before in deep learning context and could in principle be applied to any other machine learning pipeline."

Other comments and suggestions:

1. Line #149: "since CNN on single images performed poorly at predicting extrusion" – can you show this result for comparison?

Answer: This was indeed based on our very early attempt based on single time points for which we have not really documented the performance (since we rapidly stopped using this strategy). Since we cannot document this, we have decided to remove this sentence and rather justify why in principle temporal dynamics is essential to detect extrusion, line 152:

"Since cell extrusion is mostly defined by the dynamics of cell shape changes (progressive apical constriction), we decided to include temporal information by using a recurrent neural network architecture."

Moreover, as discussed above we have now documented the impact of the size of temporal window used for the prediction and showed that a reduction of the window size to two time frames drastically reduced the accuracy of the predictions.

2. Fig. 2G-H are not referenced from the main text.

Answer: Sorry if this was not clear. Actually, we did refer to this panel in the line 225 to mention that the pipeline seems to perform well at any stage of development of the pupal notum. This is now in line 248 (the panels have changed to Fig 2. E,F):

*"We observed a fairly constant error (~3 errors every 10 frames/50 minutes) suggesting that our methodology is robust at all developmental stages imaged in the notum (**Fig.2E-F**). Thus, our methodology can retrieve the vast majority of the extrusion events at any stage of development."*

3. Instead of reporting size in pixels (e.g., line #184, Fig. 2D-E, line #265) or time in frames (e.g., Fig. 2D,F, Fig. S1C, line #524), please report in microns and minutes correspondingly throughout the manuscript.

Answer: Thanks for bringing this point. We actually had quite a lot of discussion among us regarding the most relevant way to report spatial and temporal scale. Our pipeline includes a scaling inputs regarding the characteristic size of cells as well as the characteristic duration of extrusion (which can considerably vary between tissues). As such the 45 by 45px or the 10 frames can have very different absolute size/duration according to the tissue processed (could it be the pupal abdomen or the pupal notum). For this reason, we decided to stick to pixel and frames value as in essence the pipeline works with "biologically" normalised inputs. Unless the referee strongly disagrees with this point, we kept this but maked sure that we give correspondence in absolute size for the pupal notum (the most represented type of samples that we used for the training).

4. I could not find the Table legends.

Answer: Sorry about that. Indeed we forgot to include a table legends. This is now part of the Supplementary fused document.

© 2023. Published by The Company of Biologists under the terms of the Creative Commons Attribution License (https://creativecommons.org/licenses/by/4.0/).

5. Line #300: using manually annotated extrusion and division events from the dataset used for analysis is not problematic in terms of “training data contamination”?

Answer: This is a very relevant concern indeed. For the extension of the model to other tissues, we had to use available movies and we were as such limited by the quantity of data (for instance, we had a single movie of the pupal wing). To circumvent this problem, we performed manual annotation on a small part of the field of view and very limited number of events. For extrusions, out of 59 events that were used to test the performance, 2 events were also present in the re-training dataset of 28 extrusion events. Hopefully this should lead to limited data contamination.

6. I wonder how would the performance of a model that uses cell shape dynamics compared to DeXtrusion that uses the raw image data. This can be achieved without long-term tracking, rather using short trajectories (that are not so prune to mistakes) and training a model from shape features extracted from these trajectories. This would extend the scope of the study, and not necessary to support the main claims/conclusion.

Answer: This is a very good suggestion and an interesting point. We foresee that training based on geometrical features may help to generalise more easily the pipeline as it should be less sensitive to the type of protein tags and the variations of fluorescent intensities. However, it is very likely as well that it may result in less good recall if the variation of intensity provides also relevant information (e.g.: E-cad intensity tends to increase during apical constriction of extruding cells). It would be a very interesting alternative way to train the model. However, we would like to stress that one of the key points of our pipeline is to avoid segmentation. Indeed, while there are good pipelines for epithelial movie segmentation, segmenting hundreds of extrusions would still constitute a very heavy load. As such, we believe this would be out of the scope of this specific article and this strategy and could constitute yet a very interesting alternative solution to explore in the future. We now clearly propose this potential development in the discussion of the manuscript, line 377:

“Alternatively, integrating purely geometrical features in the training dataset (through segmentation of a large numbers of extrusions/divisions/SOPs and integrating skeletonised sequences in the training) may help to build a more generalist model less sensitive to the tagged protein and the variation of fluorescence intensity. While collecting enough segmented data may be time consuming, it could be a very promising alternative method to build a very generalist model. Introducing manually defined features into the neural network could also be an interesting approach to increase the network performance (Huang et al., 2022) but would require more computation to extract the hand-crafted features.”

Minor cosmetic suggested changes/typos

1. Line #41, extra space at the end of the sentence.
2. Line #58, missing “a” (“in a tissue”).)
3. Fig. 1A – mark the number of events in the histogram
4. In Tables S1 and S2 change the label “death” to “extrusion”
5. Fig. 2 legend: H → G, I→ H
6. Fig. 4 legend – the colors do not fit to the figure (green→ gray, magenta→ green, light purple→light green)
7. Line #503: “…)”

Answer: Thanks for catching these mistakes, our apologies. We have now corrected all these points.

Reviewer 2 Advance Summary and Potential Significance to Field

In this manuscript, Villars, Letort, and their team have developed a tool named DeXtrusion that utilizes neural networks to automatically detect biological events, such as cell extrusion, cell

© 2023. Published by The Company of Biologists under the terms of the Creative Commons Attribution License (https://creativecommons.org/licenses/by/4.0/).

division, and cell differentiation (SOP formation) in a confocal fluorescent video of a Drosophila epithelium. Impressively, DeXtrusion has been successful in reducing the time required for annotation by 10-fold with high precision. Moreover, DeXtrusion has demonstrated its applicability to other epithelial tissue, such as pupal wing and pupal abdomen, making it a valuable and versatile tool for the scientific community. Overall, I recommend the publication of this manuscript in Development, provided that several points, which are primarily clarifications and presentations, are addressed. I listed these points below.

Answer: Thanks for appreciating the usefulness of the pipeline and its versatility.

Reviewer 2 Comments for the Author

MAJOR:

#1) After reading the manuscript, I was initially confused about the different functions DeXtrusion and DeXNet. To avoid this confusion, I suggest that the authors make the following changes:
- In Fig. 1C, replace the wording “DeXtrusion model” with “DeXNet (model)”, if that is what is meant.
- Before diving into the details of the models (line 141), explain the relationship between DeXtrusion and DeXNet. My understanding is that DeXNet is the model used to detect the events in a cropped video, while DeXtrusion is the overall pipeline used to apply DeXNet to the entire time and space of the video. Clarifying this relationship at the outset will aid in better understanding the subsequent model descriptions.

Answer: These are very good suggestions and we agree that the distinction between DeXNet and DeXtrusion may have been a bit confusing. We have followed the suggestion of the reviewer by changing the labels in Figure 1C and explained now right from the beginning the decomposition of the pipeline between DeXNet (mostly described in Figure 1) and DeXtrusion (Figure 2). This is now mentioned in the main text as followed line 144:

“*DeXtrusion refers to the full pipeline detecting cellular events from the full movies (see below and **Figure 2**), while DeXNet refers to the neural network used to classify each cropped image sequence.*“

#2) Fig. 1C-C’.
The manuscript describes how temporal information history was incorporated using a recurrent neural network architecture. However, it is not clear in Fig.1C which part of the image indicates the temporal history.
- Similar presentation in Fig. 2A can be applied here, too.
- It is also unclear whether the "10@" in "10@45x45" refers to the time sequence.
To avoid confusion, the authors should elaborate on what this notation "10@" means. The same goes for "1@", "8@", and "64@" in Figure 1C.
- Is “10@64” before GRU in Fig. 1C supposed to be “10@1x64”?
- You may consider making the 1x64 parallelogram in Fig. 1C’ into a rectangle, like the yellow rectangle used before GRU (10@64).
- It would be helpful to add a few sentences describing CNN (Fig. 1C’) in the text.

Answer: We apologise if the figure was confusing. Indeed, initially, we did not want to give an extensive description of the convolutional neural networks which may end up being confusing. We have now modified Figure 1C,C’ by clearly indicating when we refer to time frame, pixel or other features (including convoluted pixels) and followed the suggestion for the rectangles and annotations (also in Figure 2A). We have also included in the Figure legend a more thorough description of the dimension reduction process. We hope this will make the network description more intelligible. You can find now the figure legend line 804:

*“**C-C’**: Schematic of the model architecture called DeXNet. **C**. Main model. The image sequence composed of 10 time frames (T) of 45 by 45 pixels (px) is passed first to a CNN detailed in **C’** which encodes each image of the sequence in a vector of 64 features. The resulting matrix for the sequence (10Tx64, representing 10 time points) is itself fed into a Gated Recurrent Unit (GRU) to consider temporal information. This is then passed to a dropout normalisation layer before going*

© 2023. Published by The Company of Biologists under the terms of the Creative Commons Attribution License (https://creativecommons.org/licenses/by/4.0/).

through a sequence of densely connected layers (dense). Finally, it predicts with a probability whether the input sequence is an extrusion or not. ***C'****: Detailed architecture of the encoding CNN. Each image in the sequence goes into a sequence of convolution (Con2D), Batch normalisation and max pooling (MaxPool, all this is repeated 4 times). The convolution was made using n=8 filters which are multiplied by 2 at each layer thereby increasing the first dimension (8,16,32,64) while the max pooling reduces the other dimension (45,22,11,5). Finally, this output (64x5x5) goes through a final step of max pooling encoding the image in a final vector of dimension (1x64)."*

#3) I have a different impression between Fig. 2D and Fig. 2E&F, particularly with regards to the temporal information. This could be due to the difference in the binning used in the figure. Therefore, I recommend that the author apply the same binning as used in Fig. 2D to Fig. 2F.

Answer: It is true that we used a different bin specially for the time histogram. Figure 2D represents a very limited part of the distribution focusing on the real event detection (which have limited temporal and spatial shift) while the two histograms were intended to show broader distributions (hence the difficulty to compare), which included large final bins that count all the events whose distance is out of range. We have decided to propose a novel representation by showing first the large-scale matrix of the distance (covering the full range of temporal and spatial distances) as well as the histogram next to the matrix, then showing a close-up view of the matrix (as shown previously in former Fig. 2E) and the corresponding distribution on the side. The bins now match the observations in the matrix. This helps to see the relevant events used to define the thresholds, while really documenting the full span of the distribution. We hope this will make the figure more readable (now **Figure 2 D,D'**)

MINOR:
#4) It would be helpful to describe how each event was manually annotated. For instance. Specifically, which software was used, and which time point (or duration?) was used to define each event, such as extrusion?

Answer: We apologise if this was not clear. All events were manually annotated using Fiji. For extrusion, the point of termination of apical constriction was manually clicked, for cell division, the point centered of the dividing cell just prior to cytokinesis (beginning of furrow constriction) were clicked, while for SOPs no specific time points were picked and all SOPs stage were randomly selected (from the first asymmetric division events to the late SOPs) systematically clicking in the center of the cell with the smallest apical area. These points are now clearly described in the Methods section as followed, line 437:

"Detection of cellular events (extrusions, SOPs, cell divisions and control regions) were performed manually on Fiji using dot ROIs and extracting x,y,t coordinates. For extrusion, the point of termination of apical constriction was manually clicked. For cell division, the center of the dividing cell just prior to cytokinesis (beginning of furrow constriction) were clicked. For SOPs, no specific time points were picked and all the SOPs stage were randomly selected (from the first asymmetric division events to the late SOPs) by clicking in the center of the cell with the smallest apical area."

#5) It would be helpful to give a number or range of "the temporal size of the sequences (line 166)".

Answer: Sorry for this mistake, we should have specified this. We now provide the number of frames and absolute duration while providing information about the characteristic duration of extrusion (line 146):

*"Each cropped image sequence (a series of 10 images, one every 5 minutes, of 45x45 pixels in the reference scale, representing 2-3 cells per frame) was processed through a neural network called DeXNet to estimate the presence or absence of an extrusion event (****Fig.1B****). "*

#6) Some of the terminologies were not well-defined, particularly for potential readers of the journal who may not be familiar with the specific terminology. I recommend that the authors provide more precise and clear definitions for the following words in the text:
- "loss" in Fig. 1D.

© 2023. Published by The Company of Biologists under the terms of the Creative Commons Attribution License (https://creativecommons.org/licenses/by/4.0/).

- “recall” in Fig. 3E’, S1, S2, and S3.
- “recall” and “precision”. What does “real events” (line 218) mean?

Answer: We apologise if this was confusing. We now make sure that every new term is properly introduced in the main text (including loss, recall, precision). We now define early on in the main text the terms Recall and Precision, and we have replaced the term “real events” in the recall definition by “proportion of manually annotated cellular events detected by the pipeline” (which is hopefully clearer). Loss is now defined in the Figure 1 legend.

#7) What’s the pixel resolution of the “final local probability output (line 191)”? Is it 22x22 pixels? It would be helpful to describe this in the text.

Answer: This is correct. We run DeXNet on 45x45 pixel square and use squares with 50% overlap. For each square, we obtain a probability value that we assigned to a 22 by 22px region in the center. As a result, probability values on the final prediction movie have indeed a spatial resolution of 22 pixels. We now clearly mention this in the main text (line 209):

*“This value is then allocated in the original movie in a 22x22 pixels and 5-time points regions around the centre of the cropped sequence. The final local probability output is then obtained by averaging for each pixel the probability of the different overlapping windows (in time) generating an extrusion probability map on the whole movie with a spatial resolution of 22px (**Fig. 2A,B, Movie 1**).”*

#8) It would be helpful to describe how the manual annotation was done, particularly whether the annotation included the time duration. If not, it would be useful to clarify which time point was considered as an "extrusion", "division", and "SOP".

Answer: Sorry again for the lack of clarity. As discussed above we now clearly defined in the Methods how each cellular event was manually annotated, including time consideration (termination of apical constriction for extrusion, the onset of cytokinesis for division and no specific time point for SOPs).

#9) To be accurate, “4 time frames (line 215)” could be “+/- 4 time frames”. Just for your consideration.

Answer: Thanks for pointing out this mistake, it should indeed be +/- and we have corrected the text accordingly.

#10) Microscope images in Fig. 3F are too small.

Answer: The images in Figure 3F were initially just here for illustration. However, we have now expanded them to show more clearly a couple of examples of false positives used for reinforcement.

#11) The main text didn’t explain the difference between “ext, SOP” vs “extr, SOP, div” shown in Fig. 3E-E”. Please expand the text.

Answer: Indeed, we did not specifically comment these results in the main text. We here include progressively different cellular events, first adding SOPs, then also including division. We now clearly specify this in the Figure legend.

#12) “extr” in Fig. 3E-E” could be “ext”.

Answer: Sorry about this mistake. “extr” is now systematically replaced by “ext” in all the figures and legends.

© 2023. Published by The Company of Biologists under the terms of the Creative Commons Attribution License (https://creativecommons.org/licenses/by/4.0/).

Second decision letter

MS ID#: DEVELOP/2023/201747

MS TITLE: DeXtrusion: Automatic recognition of epithelial cell extrusion through machine learning in vivo

AUTHORS: Alexis Villars, GaÃ«lle Letort, LÃ©o Valon, and Romain Levayer
ARTICLE TYPE: Techniques and Resources Article

I am happy to tell you that your manuscript has been accepted for publication in Development, pending our standard ethics checks.

Reviewer 1

Advance summary and potential significance to field

See review of initial submission

Comments for the author

My initial assessment of this article was positive. The authors have addressed my concerns and improved the manuscript. I recommend publication.

Reviewer 2

Advance summary and potential significance to field

In this manuscript, Villars, Letort, and their team have developed a tool named DeXtrusion that utilizes neural networks to automatically detect biological events, such as cell extrusion, cell division, cell differentiation (SOP formation) in a confocal fluorescent video of Drosophila epithelia. This tool should be a valuable tool for the community.

Comments for the author

The authors adequately responded to all my comments. I support this study to be published.

© 2023. Published by The Company of Biologists under the terms of the Creative Commons Attribution License (https://creativecommons.org/licenses/by/4.0/).
